# Monitoring the Spread of Multidrug-Resistant *Escherichia coli* Throughout the Broiler Production Cycle

**DOI:** 10.3390/antibiotics14010069

**Published:** 2025-01-10

**Authors:** Victor Dellevedove Cruz, Danilo Henrique Rabaçal Alves, Jamile Kellen de Souza, Maísa Fabiana Menck-Costa, Bruno Henrique Dias de Oliva, Ana Angelita Sampaio Baptista, Alexandre Oba, Fabrizio Matté, Kácio Emílio Borges Baierle, Sérgio Paulo Dejato da Rocha, Kelly Cristina Tagliari de Brito, Benito Guimarães de Brito, Gerson Nakazato, Marcio Costa, Renata Katsuko Takayama Kobayashi

**Affiliations:** 1 Department of Microbiology, Biological Sciences Center, State University of Londrina, Londrina 86057-970, Brazil; victor.dellevedovec@uel.br (V.D.C.); danilo.henriqueralves@uel.br (D.H.R.A.); jamile.kellen.souza@uel.br (J.K.d.S.); maisa.menckcosta@uel.br (M.F.M.-C.); bruno.henrique@uel.br (B.H.D.d.O.); rochaspd@uel.br (S.P.D.d.R.); gnakazato@uel.br (G.N.); 2 Avian Medicine Laboratory, Department of Preventive Veterinary Medicine, State University of Londrina, Londrina 86057-970, Brazil; anaangelita@uel.br (A.A.S.B.); mv.kacio.baierle@uel.br (K.E.B.B.); 3 Department of Zootechnics, Poultry, Production and Quality of Broiler Meat, State University of Londrina, Londrina 86057-970, Brazil; oba@uel.br; 4 Vetanco Brazil, Chapecó, Santa Catarina 89813-824, Brazil; fabrizio@vetanco.com.br; 5 Avian Health Laboratory, Veterinary Research Institute Desidério Finamor, Agricultural Diagnosis and Research Department, Secretariat of Agriculture Livestock Rural and Development, Eldorado do Sul 90990-000, Brazil; kellybritofepagro@gmail.com (K.C.T.d.B.); benitobrito@gmail.com (B.G.d.B.); 6 Département de Biomédecine Vétérinaire, Faculté de Médecine Vétérinaire, Université de Montréal, Montreal, QC H3C 3J7, Canada

**Keywords:** monitoring, antimicrobial resistance, virulence genes, one health, poultry

## Abstract

The extensive use of antimicrobials in broiler production is changing the bird microbiota, fostering drug-resistant bacteria, and complicating therapeutic interventions, making the problem of multidrug resistance global. The monitoring of antimicrobial virulence and resistance genes are tools that have come to assist the breeding of these animals, directing possible treatments as already used in human medicine and collecting data to demonstrate possible dissemination of multidrug-resistant strains that may cause damage to industry and public health. This work aimed to monitor broiler farms in southern Brazil, isolating samples of *E. coli* and classifying them according to the profile of resistance to antimicrobials of interest to human and animal health. We also monitored the profile of virulence genes and conducted an epidemiological survey of possible risk factors that contribute to this selection of multidrug-resistant isolates. Monitoring was carried out on farms in the three southern states of the country, collecting samples of poultry litter, cloacal swabs, and beetles of the species *Alphitobius diaperinus*, isolating *E. coli* from each of these samples. These were evaluated by testing their susceptibility to antimicrobials of animal and human interest; detecting whether the samples were extended-spectrum β-lactamase enzyme (ESBL) producers; and when positive, selected for genotypic tests to identify resistant genes (CTX-M, TEM, and SHV) and virulence. Among the antimicrobials tested, enrofloxacin and ciprofloxacin demonstrated some of the highest frequencies of resistance in the isolated strains, with significant statistical results. The use of these antimicrobials increased the likelihood of resistance by over three times and was associated with a 1.5-fold higher probability of multidrug resistance. Of all isolates, 95% were multidrug-resistant, raising concerns for production and public health. Among 231 ESBL-positive samples, the CTX-M1 group predominated.

## 1. Introduction

The agricultural sector in Brazil has played a significant role in the country’s economy over recent decades, evidenced by a production of 14.5 million tons of poultry meat in 2023. Notably, the southern region of the country is responsible for over 60% of national poultry slaughter [1]. It is the sector that has invested the most in the intensification of its production, aiming to achieve key goals such as faster production and lower costs, becoming one of the largest industries in the world in terms of production and quality [2].

For such high production, a large number of antimicrobials is used in poultry farming, and their use tends to create selective pressure on the bacteria present in that environment, increasing the occurrence of antimicrobial resistance [3]. Resistance in bacteria occurs naturally, but the increased use of antimicrobials has resulted in the elimination of susceptible strains, fostering the selection and proliferation of resistant strains [4]. 

One of the widely used classes of antimicrobials is β-lactams [5], prescribed in both human medicine and animal production. Consequently, an escalation of resistance has been reported due to the administration of these antimicrobials, with the main underlying mechanism being the presence of extended-spectrum β-lactamases (ESBLs) encoded by genes such as *TEM*, *SHV*, and *CTX-M* [6].

Production animals can harbor resistant pathogenic bacteria, and their genes can be transferred to non-pathogenic bacteria present in the microbiota, spreading these genes throughout the production chain and subsequently to humans through food consumption [7,8].

*Escherichia coli*, belonging to the family *Enterobacteriaceae,* possesses a facultative anaerobic metabolism; lacks spore formation; and ferments various sugars such as lactose, sucrose, and glucose. Its presence in water and food can serve as an indicator of fecal contamination and is also used as a marker for antimicrobial resistance [9,10,11].

*E. coli* can exhibit numerous virulence factors that enable it to cause extraintestinal infections, including avian colibacillosis [12]. The most frequently described virulence factors include the expression of adhesins, enterotoxins, iron acquisition systems in the bloodstream, resistance to serum antibodies, a host complement system with cytotoxic necrotizing factor, and hemolysin [13]. All factors contribute to the survival and rapid progression of the disease [14].

The aim of this study was to monitor virulence genes and antimicrobial resistance as well as to indicate relevant zootechnical indices related to production and associated with resistance in broiler farms and breeder farms in southern Brazil. This was achieved through phenotypic and genotypic tests, generating important epidemiological data for tracking current resistance in the country.

## 2. Results

### 2.1. Characteristics of Farms and E. coli Isolates

In this study, a total of 28 distinct farms located in the southern region of Brazil were analyzed, and three breeder farms were also analyzed. A collection of 751 strains of *E. coli* was isolated from cloacal swab samples of the chickens (250), beetles (252), and poultry litter (249). 

The Information Sheet gathered important data for both production-related aspects and the research conducted in this study. Among the 28 farms under investigation, the number of chickens per poultry house ranged from 16,000 to 34,600, with ages ranging from one day to 34 days. Additionally, all farms reported treating the water supplied to the birds with chlorine. Among these farms, seven stated the absence of any other activities or elements surrounding their poultry houses, while the rest reported the presence of various activities, such as pig farming, beef cattle farming, dairy farming, free-range chickens, and beekeeping in the proximity.

Regarding the treatment of poultry litter prior to the birds’ entrance into the houses, seven farms reported refraining from any treatment, while the remaining farms utilized fermentation, lime, and other routine techniques for litter management. Notably, it is pertinent to highlight that six farms that reported not treating their litter prior to the birds’ entrance exhibited multi-drug resistant (MDR) *E. coli* in all the analyzed samples and strains, including poultry litter, beetles, and cloacal swabs.

The number of poultry litter reutilizations varied from farms that used newly placed litter to others that had more than 23 reutilization cycles. Regarding the batches from which the samples were collected, 79% contained antimicrobials, such as florfenicol, enrofloxacin, amoxicillin, virginiamycin, enramycin, ciprofloxacin, tilmicosin, and sulfamethoxazole + trimethoprim.

Most of the antimicrobials were used for animal treatment. However, in four farms (14, 15, 16, 17), there was a notable escalation in antimicrobial usage, wherein preventive administration, growth promotion, and animal treatment occurred concomitantly within a single production cycle. Due to this extensive antimicrobial usage in these specific farms, all strains (100%) showed multidrug resistance, with frequencies ranging from 40% to 100% in terms of positivity for ESBL enzyme production.

### 2.2. Phenotypic Antimicrobial Resistance

**Figure 1:**
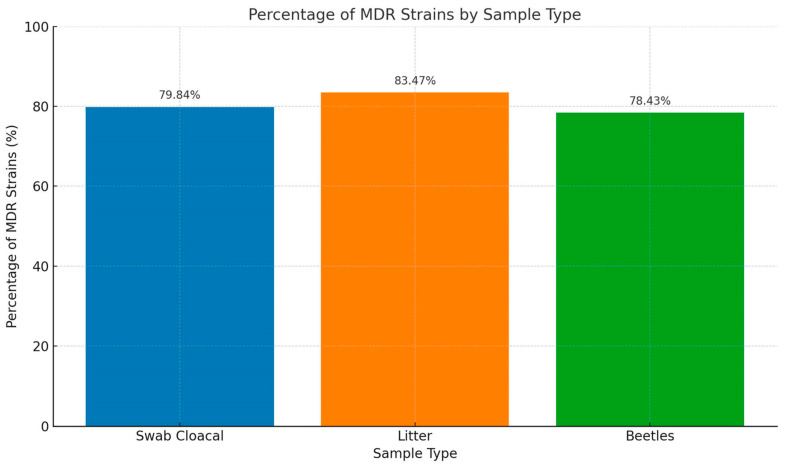
Percentage of MDR by sample.

Out of the 751 isolates, 713 (95%) were classified as multidrug-resistant (MDR), indicating their resistance to three or more distinct classes of antimicrobials. All the antibiogram results described are in Table S1. Notably, all isolates that tested positive for ESBL production were also identified as multidrug-resistant. Among the 28 analyzed farms, 20 farms showed a profile of multidrug resistance in all their isolates (Table 1). The farm with the lowest percentage of MDR (66%) was farm four, which presented noteworthy characteristics based on the collected information. Importantly, no antimicrobials were used throughout the production cycle until the sampling, and the litter underwent prior treatment with lime before bird placement. Figure 1 shows a graph with the percentages of multidrug-resistant bacteria by sample collected from the farms.

**Figure 2:**
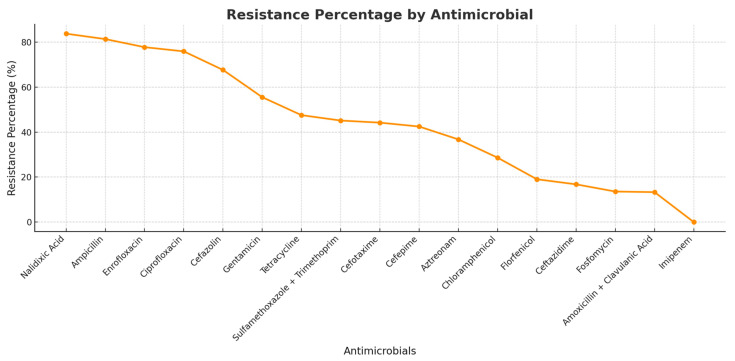
Resistance percentages.

None of the isolates displayed sensitivity or resistance to all tested antimicrobials, and there was no strain found to be resistant to the carbapenem antimicrobial imipenem. Among the total strains analyzed, resistance to the quinolone class was particularly notable, with 83.5% showing resistance to nalidixic acid, 81.5% to ampicillin, 77.5% to enrofloxacin, and 75.5% to ciprofloxacin. These and other results are shown in Figure 2.

A total of 79% of the isolates showed resistance to more than three classes of antimicrobials in the breeder farms. Among the *E. coli* isolates, 56% were ESBL producers.

### 2.3. Detection of ESBL Genes (bla_CTX-M_; bla_TEM_; bla_SHV_) and Virulence Genes

All strains were subjected to the disk diffusion test and double disk synergy test. Subsequently, strains showing a positive profile for ESBL enzyme production were selected for further genotypic tests. Out of the total isolates, 231 (30.7%) strains tested positive for ESBL enzyme production. All positive strains were analyzed for the presence of *bla*_CTX-M_, *bla*_TEM_, and *bla*_SHV_ resistance genes, as outlined in the methodology. Additionally, in accordance with Johnson et al. (2008) [15], this study also encompassed the analysis of five virulence genes, which were identified as the minimum predictors of APEC.

In the comprehensive analysis of the CTX-M group frequencies, CTX-M-1 was the most identified group, with a frequency of 37% (85), followed by CTX-M-9 at 26% (60), CTX-M-2 at 18% (43), and CTX-M-8 at 15% (34). Interestingly, the CTX-M-25 group was not detected in any of the analyzed strains, as shown in Table 2 and Table 3. Regarding the TEM gene, 39.4% (91) of strains tested positive, while in the SHV gene, 17.7% (41) were positive. Among the 91 strains positive for the TEM group, 35 (38.5%) also carried the *bla*_CTX-M_ gene.

In the breeder flocks studied, 60 (66%) of the *E. coli* isolates carried *bla*_CTX-M_ genes (CTX-M1, CTX-M2, CTX-M8, and CTX-M9 groups). Of those that presented with the genes, 48 (81%) exhibited *bla*_CTX-M1_, followed by *bla*_CTX-M2_ (30%), *bla*_CTX-M8_ (18%), and *bla*_CTX-M9_ (12%). Some strains presented more than one gene related to the CTX-M group.

Regarding the presence of virulence genes, the most frequently detected gene in the overall results of the study was *ompT*, found in 81 strains (35%). Following this, *hlyF* was found in 76 strains (34%), *iss* in 69 strains (30%), *iroN* in 65 strains (28%), and *iutA* in 63 strains (27%). Furthermore, 101 strains (43.7%) possessed at least two of the investigated genes, and 14.3% of the strains carried all five genes, as illustrated in Table 2.

### 2.4. Multivariate Logistic Regression

In this study, a multivariate logistic regression analysis was performed utilizing a 95% confidence interval. The results revealed significant association between various factors and antimicrobial resistance patterns among the isolated strains. All the results are in Table 4, Table 5, Table 6, Table 7, Table 8 and Table 9.

Strains originating from chicken litter exhibited 1.91 times higher odds (OR 1.91, *p* ≤ 0.05) of being classified as multidrug-resistant (MDR). Additionally, strains resistant to fosfomycin displayed 1.66 times higher odds of producing ESBL enzymes compared to strains with other types of resistance (OR 1.66, *p* ≤ 0.05). Furthermore, isolated strains from chicken litter showed 1.93 times higher odds of being resistant to ciprofloxacin compared to other antimicrobials (OR 1.93, *p* ≤ 0.05), while in the case of cloacal swabs, the odds were 1.66 times higher (OR 1.66, *p* ≤ 0.05).

When assessing the relationship between the variable of MDR strains and the use of antimicrobials in the poultry production, it was observed that the odds of strains being MDR in these farms were 1.5 times higher (OR 1.5, *p* ≤ 0.05). Moreover, the use of enrofloxacin in production was associated with 3.58 times higher odds of isolating strains resistant to this antimicrobial (OR 3.58, *p* ≤ 0.05), and for ciprofloxacin, it was 6.36 times higher (OR 6.36, *p* ≤ 0.05). These findings underscore the impact of antimicrobial usage in poultry production on the emergence of antimicrobial resistance in the isolated strains.

## 3. Discussion

The AgroPrevine initiative, as per IN Nº 41 dated 23 October 2017 [16], is based on the One Health concept, encompassing activities and strategic interventions aligned with epidemiological studies, surveillance, and monitoring of antimicrobial resistance. In this study, a comprehensive analysis of farms was undertaken, revealing that 91% of the strains were classified as multidrug-resistant, with more than 30% of the strains exhibiting ESBL-producing characteristics, indicating alarming epidemiological results in the production setting. The Centers for Disease Control and Prevention (CDC) [17] described ESBL strains as serious threats in their 2019 report, estimating that ESBL strains caused over 197,000 hospitalizations in 2017, incurring a healthcare system cost of over 1 billion dollars.

The four farms (Farms 14, 15, 16, 17) that utilized antimicrobials as growth promoters, for prophylaxis, and as treatment, showed strains resistant to the specific antimicrobials used, including amoxicillin and ciprofloxacin, which were administered for animal treatment. This practice can exert selective pressure on bacteria in poultry production [18], leading to difficulties in subsequent treatments and potentially promoting the selection of bacteria producing antibiotic resistance enzymes such as ESBL.

This study also conducted logistic regression analyses with a 95% confidence interval, revealing that strains originating from the poultry bedding had 1.91 times higher odds of being MDR compared to other sample sources, emphasizing the importance of effective bedding management for ensuring safe production and preventing the dissemination of genes and pathogenic bacteria to subsequent flocks. The implementation of composting and the use of lime in bedding have been shown to reduce organic residues and significantly decrease pathogenic microorganisms for the birds [19]. These practices can play a crucial role in mitigating antimicrobial resistance in poultry production, contributing to more sustainable and safer agricultural practices. 

The statistical analysis conducted in this study revealed a significant relationship between multidrug resistance and the use of antimicrobials in poultry production. The experimental findings corroborate the work of Han et al. (2020) [20], who analyzed phenotypic and genotypic profiles of *E. coli* strains before and after the use of antimicrobials in broiler production. They observed that strains exposed to antimicrobials during production exhibited 1.5 times higher odds of being MDR.

Our study also found a statistically significant association between the use of ciprofloxacin in the poultry farm and strains showing 6.36 times higher odds of resistance to this antimicrobial compared to farms that did not use it in production. A similar observation was made for enrofloxacin, with strains exhibiting 3.58 times higher odds of resistance when this antimicrobial was employed for animal treatments compared to farms that did not use it.

Furthermore, a study conducted by Gazal et al. (2015) [21] analyzed poultry bedding after composting and reported that most of the isolates displayed a low frequency of virulence and antimicrobial resistance genes. In our study, poultry farms that utilized new bedding for their animals at the time of sampling showed low frequency of MDR strains. On average, the strains were sensitive to six out of the seventeen tested antimicrobials, in contrast to farms that did not treat their bedding, which showed sensitivity to only two antimicrobials. 

Among the 751 isolated strains, 30.7% tested positive for ESBL production, which represents a lower frequency compared to the findings reported by Gazal et al. (2021) [10] in a longitudinal monitoring study conducted in the southern region of Brazil, where 67% of ESBL-producing isolates were identified. Conversely, the multidrug resistance observed in this study was higher, with 95% of strains classified as MDR, exceeding the 80% of MDR isolates reported by Cyoia et al. (2019) [22] in commercialized chicken carcasses. This disparity may indicate that these multidrug-resistant and ESBL-producing bacteria are not limited solely to the farms but can persist in the final product, i.e., chicken meat, posing a potential transmission risk to humans.

Remarkably, resistance to antimicrobials was particularly prominent in the quinolone class, with 75% of the samples showing resistance to ciprofloxacin. Similar results were observed in the study by Nguyent et al. (2017) [23], which reported 73% resistance to ciprofloxacin in avian-origin *E. coli* strains in Vietnam. The high percentage of ciprofloxacin resistance in our study can be justified by its utilization in the farms, with six farms reporting its use at the time of sample collection, and it is plausible that this antimicrobial was also used in other batches. A similar pattern applied to resistance to enrofloxacin (77.5%), with seven farms reporting its use in the batch. Hachesoo et al. (2017) [24] reported 80% resistance to quinolones in a study conducted in Iran, which is consistent with the findings of Azizpour and Saeidi (2018) [25], who reported 77% resistance.

Ciprofloxacin and enrofloxacin, both belonging to the quinolone class, are widely used in the field for the treatment and prophylaxis of animals. In our study, samples isolated from bedding had 1.93 times higher chances of being resistant to ciprofloxacin, while samples isolated from cloacal swabs had 1.66 times higher chances. Conversely, the samples from beetles did not show statistically significant resistance.

Through PCR-based resistance gene detection, we identified 231 strains as ESBL producers, with the most frequently detected group being CTX-M1, found in 38% (86) of the isolates. This differs from the study by Gazal et al. (2021) [10], who conducted a longitudinal study in broiler farms in Southern Brazil (2016–2018) and reported CTX-M2 as the most prevalent gene (56%).

Such discrepancies suggest a possible shift in the prevalence of *bla*_CTX-M_ genes in the country, as observed in a more recent study by Menck-Costa et al. (2022) [9]. In their study, CTX-M9 was not detected, which differs from our findings, as *bla*_CTX-M9_ was detected in 24% (60) of the strains. The CTX-M9 gene has been widely reported in older studies, particularly in Europe in countries such as Spain and the United Kingdom, and is mainly associated with human infections, suggesting a possible link between animal-derived products and the reservoir of these genes [26]. The group CTX-M25 gene was not detected in either study.

Among the 91 strains positive for the *bla_TEM_* gene, 35 of them also carried genes from the CTX-M group. The coexistence of different β-lactamases in the same isolates has been reported in studies by Li et al. (2016) [27] and He et al. (2013) [28]. The most common combination observed in this study was the presence of *bla_CTX-M_* and *bla_TEM_* genes together, while the combination of all three genes (*bla_CTX-M_*, *bla_TEM_*, and *bla_SHV_*) was not observed in the same isolate.

Analyses by Koga et al. (2019) [11], Gazal et al. (2021) [10], and Cyoia et al. (2019) [22] demonstrated a higher prevalence of CTX-M2, while our study and that of Menck-Costa et al. (2022) [9] suggest a possible shift in the resistance gene profile for the CTX-M group in the country. The acquisition and dissemination of these genes may be facilitated through the interaction of microbiota bacteria with environmental bacteria [29], which may already be present in the production system and persist due to potential failures in litter management, housing conditions, and improper antimicrobial use. This highlights the importance of monitoring and surveillance in broiler farms to comprehend the current conditions of broiler production.

Some virulence genes found in avian pathogenic *Escherichia coli* (APEC) related to iron uptake in the host system, i.e., *iutA* and *iroN*, were analyzed in this experiment, with percentages of 27% (63) and 28% (65), respectively. The acquisition of iron by bacteria is highly valuable due to the low concentration of iron in the sites where ExPEC typically infects its hosts, making it an important characteristic for highly virulent strains [15]. 

Additionally, the *iss* gene encodes a virulence factor that enables bacteria to evade the host immune system by blocking the binding of the complement system complex with the bacterial surface, preventing bacterial lysis [15]. This gene was observed in 69 of the analyzed strains. These genes collectively indicate that bacteria in the production system are becoming increasingly virulent and resistant.

In this study, all strains possessing at least one virulence gene were classified as MDR, with 57% of them having at least two virulence genes, fulfilling the minimum criteria for classification as APEC if isolated from lesions [15].

This study focused on *Escherichia coli* due to its significant role in antimicrobial resistance and its impact on poultry production. Although resistance in other bacteria is undoubtedly significant, *E. coli* provides a strong starting point for understanding broader resistance patterns. Expanding to other bacterial species could be a valuable direction for further research and complement these findings.

Considering the high global demand for Brazilian poultry, meticulous attention to every detail of our production process, including proper management of animals and facilities, becomes imperative to ensure safer production for consumers in over 130 countries. This approach provides a wealth of information that can subsequently aid in more efficient animal management practices.

Farmers play a vital role in preventing antimicrobial resistance. Educating farm workers about the rational use of antimicrobials and biosecurity best practices is the first step to preventing antimicrobial resistance in poultry production. Farmers also should prioritize biosecurity by restricting farm access, disinfecting equipment, pest control (beetles), managing the poultry litter more regularly, and preventing contact with wild animals. Along with responsible antibiotic use under veterinary guidance and monitoring the results of antibiograms conducted regularly on farms, treatments should be directed toward more effective and precise solutions for improving animal health and preventing significant economic and production losses.

Multidrug resistance in *E. coli* associated with resistance and virulence factors poses a significant challenge at all stages of production, particularly in broiler farms. Continuous monitoring and surveillance should be implemented on farms as a fundamental tool for detection, as they are considered the first step towards improving and regressing the current production situation. 

## 4. Materials and Methods

### 4.1. Study Period and Location

The study was conducted from 2021 to 2023. Samples were collected from various locations in the states of Rio Grande do Sul, Santa Catarina, and Paraná. Subsequently, all collected samples were processed and analyzed at the Basic and Applied Microbiology Laboratory (NIP3) of the State University of Londrina (UEL), located in Londrina, Paraná.

### 4.2. Characteristics of the Farms

The samples were collected from a total of 28 broiler chicken farms and 3 breeder farms. For each farm, a comprehensive farm information sheet was administered containing questions concerning the poultry house dimensions, production management practices, the current bird population within the house, litter management methods, frequency of litter reuse, and history of antimicrobial use, among other relevant parameters (Table 1).

### 4.3. Sample Collection Procedure

Samples were systematically obtained from various sources within all farms, including chicken, poultry litter, and beetles of the species *A. diaperinus*, commonly called darkling beetles. To ensure sample integrity, all collected samples were refrigerated throughout the collection process, with a maximum time interval of 48 hours between collection and subsequent processing.

A total of 20 cloacal samples were carefully collected from each analyzed poultry house and 10 from the breeder farms. Swabs used for sample collection were equipped with absorbent wooden shafts and were stored in a Cary Blair medium. The selection of birds for sampling was conducted randomly, encompassing individuals from various locations within the poultry house. For processing, the swabs were incubated in sterile Buffered Peptone Water at 37 °C (±1 °C) for 18 to 24 h.

The poultry litter was collected using two pairs of shoe covers, which were pre-moistened with a 1% Buffered Peptone Water solution. Each pair of shoe covers was used to traverse half of the poultry house’s length, and subsequently, they were carefully placed in sterile bags and promptly refrigerated. The shoe covers were later incubated with 90 mL of sterile Buffered Peptone Water at 37 °C (±1 °C) for 18 to 24 h.

Approximately 50 insects were collected per poultry house and the breeder farms, whenever they were available. During the processing phase, the beetles were frozen and then washed by immersion in sterile PBS (Phosphate Buffered Saline) for 10 minutes. Afterward, they were transferred to a solution of 70% alcohol for 10 minutes, removed, and placed on a sterile petri dish. Once dried, the beetles were macerated and then incubated in sterile Buffered Peptone Water at 37 °C (±1 °C) for 18 to 24 h [30].

### 4.4. Isolation of E. coli

After incubation, the samples were streaked onto MacConkey agar plates and subsequently incubated at 37 °C (±1 °C) for 18 to 24 h. Following growth, a minimum of ten colonies per sample plate were meticulously selected for screening, employing a battery of biochemical tests, including Triple Sugar Iron (TSI); Sulfite Indole Motility (SIM); Simmons’ citrate agar; and urea, lysine, and sorbitol broths. Colonies exhibiting a positive profile for *E. coli* (TSI: acid/acid, no H_2_S production; SIM: indole-positive; citrate and urea: negative; lysine and sorbitol: positive) were stored in Brain Heart Infusion (BHI) broth supplemented with 30% glycerol and refrigerated at −20 °C.

As a result, a total of 30 strains were isolated from each farm, with 10 strains from each type of collected sample (i.e., poultry litter, birds, and beetles) whenever available.

### 4.5. Antimicrobial Susceptibility Testing

**Figure 3:**
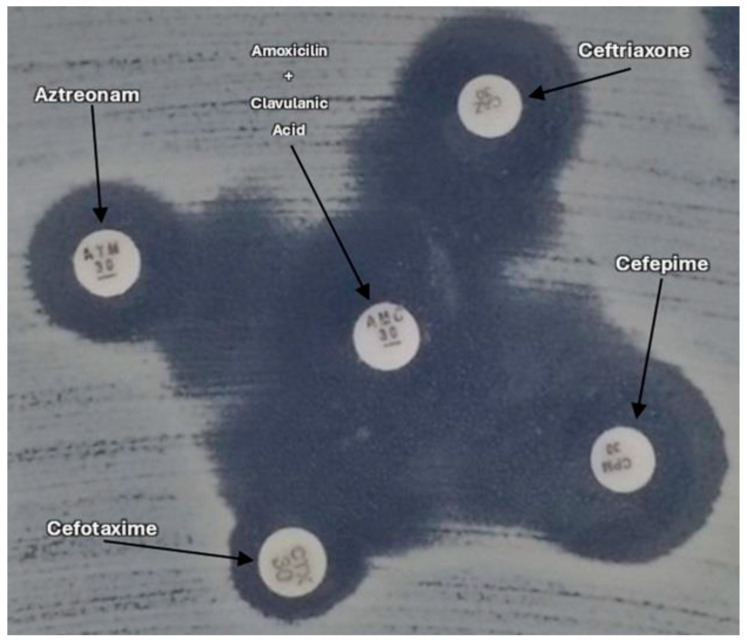
Double disk synergy test.

Antimicrobial susceptibility testing was conducted using the disk diffusion technique following the guidelines outlined by the Clinical and Laboratory Standards Institute [31]. Seventeen antimicrobials discs (Oxoid, Thermo Fisher Scientific, Waltham, MA, USA) from seven different classes were utilized as follows: β-lactams, including amoxicillin-clavulanic acid (AMC, 20/10 μg), cefazolin (CFZ, 30 μg), ampicillin (AMP, 10 μg), cefotaxime (CTX, 30 μg), cefoxitin (CFO, 30 μg), cefepime (FEP, 30 μg), aztreonam (ATM, 30 μg), and imipenem (IPM, 30 μg); sulfonamides, including sulfamethoxazole-trimethoprim (SUT, 1.25/23.75 μg); tetracyclines, including tetracycline (TET, 30 μg); quinolones, including ciprofloxacin (CIP, 5 μg), enrofloxacin (ENR, 10 μg), gentamicin (GEN, 10 μg), and nalidixic acid (NAL, 30 μg); phenicols, including chloramphenicol (CLO, 30 μg) and florfenicol (FLF, 30 μg); fosfomycin (FOS, 200 μg); and aminoglycosides, including gentamicin (CN, 10 μg). Additionally, the phenotypic ESBL detection test was performed using the double disk synergy test (Figure 3), with disks containing the inhibitor clavulanic acid (AMC, 20/10 μg).

### 4.6. DNA Extraction

The strains were cultured in 1 mL of Luria Bertani Broth (LB) (Difco R, Sparks, NV, USA) at 37 °C for 24 h. After growth, 200 μL of the culture was subjected to boiling at 100 °C for 10 min in a water bath, followed by centrifugation at 12,000× *g* for 6 min. Finally, the supernatant containing the extracted DNA was stored at −20 °C for further analysis.

### 4.7. Detection of ESBL Genes and Virulence Genes for Potential Avian Pathogenic Escherichia coli (APEC)

All *E. coli* strains identified as ESBL-positive in the phenotypic double disk synergy test were subjected to PCR testing to investigate the presence of ESBL resistance genes. The target genes searched were categorized into groups: CTX-M-1, CTX-M-2, CTX-M-8, CTX-M-9, and CTX-M-25 [32]; TEM; and SHV [33]. The description of these genes can be found in Table 10.

**Figure 4:**
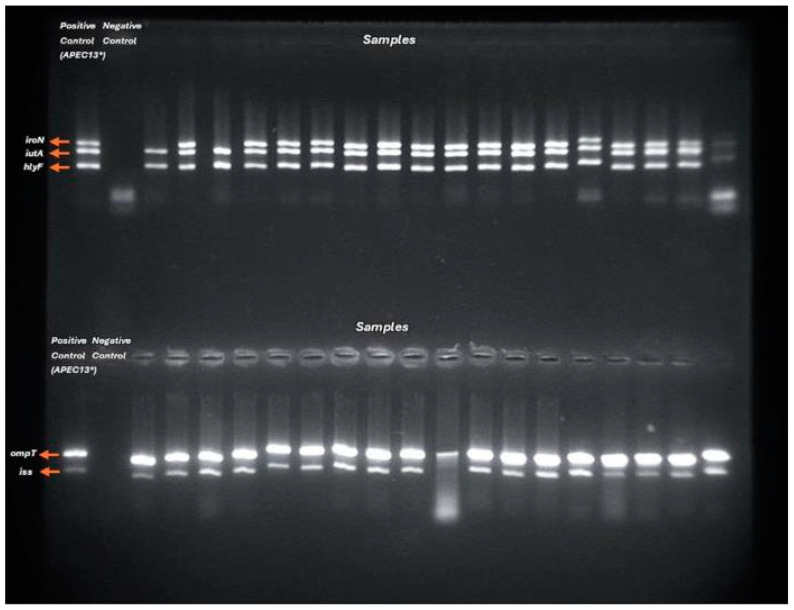
Pentaplex PCR test result.

Furthermore, five genes encoding virulence factors for extraintestinal pathogenic *Escherichia coli* (ExPEC) were investigated in ESBL-positive strains identified in the phenotypic test. The selected genes were *iutA*, *hlyF*, *iss*, *iroN*, and *ompT* [15], and the Pentaplex PCR test was performed, as shown in Figure 4. Our positive control was provided by Luís Eduardo S. Gazal, isolated in the study by Gazal (2015) [21].

### 4.8. Statistical Analysis

The statistical analysis was conducted using R software, version 3.5.1. To evaluate the relationship between the studied variables, a multivariate logistic regression analysis was performed, and the odds ratio (OR) with a 95% confidence interval (CI) was calculated. A significance level of *p* ≤ 0.05 [34] was adopted to determine the set of information that best explains the association between risk factors and the occurrence of ESBL-producing *E. coli* and multidrug resistance.

## 5. Conclusions

Farms from the three southern states of Brazil were analyzed, and a total of 751 *E. coli* isolates were obtained from poultry litter, cloacal swabs, and beetles found in the litter. Among the most used antimicrobials in production were sulfamethoxazole, enrofloxacin, ciprofloxacin, and amoxicillin, for which high levels of bacterial resistance were observed.

Enrofloxacin and ciprofloxacin were among the antimicrobials to which the bacteria exhibited the highest resistance rates, with statistically significant results. The use of these antimicrobials increased the likelihood of bacterial resistance in the samples by more than three times compared to farms that did not use them. Additionally, a statistical association was observed between multidrug resistance and antimicrobial use, as samples were 1.5 times more likely to be multidrug-resistant when antimicrobials were used in production.

Of the total isolates, 95% were classified as multidrug-resistant, an alarming figure for both production and public health. Among the 231 samples positive for ESBL enzyme production, the CTX-M1 group stood out compared to the other groups evaluated, TEM and SHV. This differed from both recent and older studies, which indicated that the *bla*_CTX-M2_ gene was the most prevalent. Various virulence genes were detected in the samples, with all virulent isolates showing multidrug resistance.

All these epidemiological data, combined with the production’s zootechnical indices, highlight the importance of proper poultry management and facility maintenance. These are critical points in production that can lead to serious issues for the animals if not carefully monitored.

## Figures and Tables

**Table 1:** Percentage of MDR (multidrug-resistant), ESBL (extended-spectrum beta-lactamase), poultry’s age, and antimicrobials used.

Farms	(%) MDR	(%) ESBL	Poultry Age (Days)	Antimicrobials Used	Litter Treatment
G1	93.83	14	3	SUT	Fermentation
G2	91.67	58	3	SUT	Fermentation + calcium
G3	87.50	18.75	5	-	-
G4	66.67	27	4	-	-
G5	100	42	5	SUT	Calcium
G6	100	20	5	FLO	Fermentation
G7	100	10	7	SUT	Calcium + disinfectant
G8	100	25	5	SUT	Calcium
G9	100	36.60	10	EN	Calcium
G10	83.33	66.66	3	SUT	Fermentation
G11	100	36.66	10	-	Calcium
G12	100	34.37	13	-	-
G13	100	40	5	-	Fermentation
G14	100	77.27	33	AMO/VIR/ENR/EN/CIP	-
G15	100	40	20	AMO/VIR/ENR/EN/CIP	-
G16	100	60.71	20	AMO/VIR/ENR/EN/CIP	-
G17	100	100	25	AMO/VIR/ENR/EN/CIP	Calcium
G18	86.66	53.33	40	VIR	Chicken litter replacement
G19	100	44,82	15	EN/CIP	Calcium
G20	100	63.33	14	EN	Calcium + disinfectant
G21	100	75	8	OR	Calcium + disinfectant
G22	100	70	6	-	Chicken litter replacement
G23	100	66.66	12	SUT	Formaldehyde
G24	100	50	43	-	Chicken litter replacement
G25	100	21.21	40	FLO	Chicken litter replacement
G26	71.43	57.14	21	-	Chicken litter replacement
G27	100	55	43	TYL	Chicken litter replacement
G28	95.83	41,38	40	EN/NEO	-

Sulfamethoxazole—SUT; florfenicol—FLO; enrofloxacin—EN; amoxicillin—AMO; virginamycin—VIR; enramycin—ENR; ciprofloxacin—CIP; oregano—OR; tylosin—TYL; neomycin—NEO.

**Table 2:** Variation in the number of virulence genes per total number of strains.

Virulence Genes	Strain Number (%)
0	130 (56.3)
1–2	25 (10.8)
3–4	43 (18.6)
5	33 (14.3)
Total	231 (100)

**Table 3:** Relationship between MDR and sample origin.

Sample Origin	Crude OR (95% CI)	Adjusted OR (95% CI)	*p* (LR Test)
Litter	3.24 (1.67, 6.3)	1.91 (1.35, 2.71)	<0.001
Cloacal Swab	0.72 (0.44, 1.19)	1.15 (0.86, 1.54)	0.359
Beetle	0.51 (0.31, 0.86)	0.87 (0.65, 1.17)	0.359

**Table 4:** Relationship between strains resistant to fosfomycin and ESBL.

Antimicrobial	Crude OR (95% CI)	*p* (LR Test)
Fosfomycin	1.66 (1.28, 2.17)	<0.001

**Table 5:** Relationship between strains from chicken litter and resistance to ciprofloxacin.

Chicken Litter	Crude OR (95% CI)	Adj. OR (95% CI)	*p* (LR Test)
Aztreonam	1.09 (0.8, 1.49)	1.38 (1, 1.9)	0.05
Cefotaxime	0.93 (0.69, 1.26)	0.7 (0.51, 0.95)	0.025
Ciprofloxacin	1.69 (1.17, 2.43)	1.93 (1.42, 2.64)	<0.001
Tetracycline	1.43 (1.06, 1.92)	1.26 (1.04, 1.52)	0.016
Nalidixic acid	0.93 (0.62, 1.38)	0.56 (0.4, 0.8)	<0.001

**Table 6:** Relationship between strains from cloacal swabs and resistance to ciprofloxacin.

Cloacal Swab	Crude OR (95% CI)	Adj. OR (95% CI)	*p* (LR Test)
Cefepime	0.85 (0.63, 1.15)	0.78 (0.62, 0.98)	0.035
Cefazolin	1.29 (0.94, 1.78)	1.46 (1.15, 1.86)	0.002
Gentamycin	1.13 (0.84, 1.53)	1.19 (0.98, 1.44)	0.082
Ciprofloxacin	1.68 (1.17, 2.42)	1.66 (1.31, 2.11)	<0.001
Ampicillin	0.63 (0.43, 0.91)	0.76 (0.58, 0.99)	0.043

**Table 7:** Relationship between MDR and previous treatment/antimicrobial use.

Factor	Crude OR (95% CI)	Adjusted OR (95% CI)	*p* (LR Test)
Previous treatment	0.58 (0.31, 1.09)	0.66 (0.48, 0.91)	0.009
Antimicrobial use	1.86 (1.12, 3.07)	1.5 (1.15, 1.97)	0.003

**Table 8:** Relationship between use of enrofloxacin and isolating strains resistant to this antimicrobial.

Use	Crude OR (95% CI)	Adj. OR (95% CI)	*p* (LR Test)
Amoxicillin + C. acid	0.86 (0.59, 1.25)	0.6 (0.44, 0.83)	0.001
Aztreonam	2.63 (1.9, 3.65)	1.8 (1.16, 2.81)	0.007
Cefotaxime	1.99 (1.45, 2.75)	0.34 (0.2, 0.57)	<0.001
Gentamycin	0.54 (0.39, 0.75)	0.78 (0.61, 1)	0.053
Fosfomycin	3.61 (2.35, 5.54)	1.49 (1.01, 2.22)	0.044
Enrofloxacin	31.47 (9.92, 99.79)	3.58 (1.96, 6.56)	<0.001

**Table 9:** Relationship between resistance to associated antimicrobials and ciprofloxacin use.

Antibiotic	Crude OR (95% CI)	Adjusted OR (95% CI)	*p* (LR Test)
Amoxicillin/clavulanate	0.71 (0.44, 1.14)	0.61 (0.43, 0.87)	0.006
Cefazolin	1.81 (1.15, 2.85)	1.69 (1.07, 2.65)	0.021
Cefotaxime	1.58 (1.08, 2.33)	0.51 (0.33, 0.8)	0.004
Ciprofloxacin	24.95 (6.1, 101.96)	6.36 (2.94, 13.78)	<0.001
Nalidixic acid	28509842.59 (0, Inf)	187.35 (5.26 × 10^126^)	<0.001

**Table 10:** Primers used in PCR for detection of resistance genes of the CTX-M groups.

Genes	Sequence (5′→3′)	PCR Product Size (bp)	Reference
*bla*-_CTX-M-1_	AAAAATCACTGCGCCAGTTC AGCTTATTCATCGCCACGTT	415	(Woodford et al., 2005) [32]
*bla*-_CTX-M-2_	CGACGCTACCCCTGCTATT CCAGCGTCAGATTTTTCAGG	552	
*bla*-_CTX-M-8_	TCGCGTTAAGCGGATGATGC AACCCACGATGTGGGTAGC	666	
*bla*-_CTX-M-9_	CAAAGAGAGTGCAACGGATG ATTGGAAAGCGTTCATCACC	205	
*bla*-_CTX-M-25_	GCACGATGACATTCGGG AACCCACGATGTGGGTAGC	327	
*bla-_TEM_*	TTGGGTGCACGAGTGGGTTA TAATTGTTGCCGGGAAGCTA	504	(Arlet; Philippon, 1991) [33]
*bla-_SHV_*	TCGGGCCGCGTAGGCATGAT AGCAGGGCGACAATCCCGCG	626	

## Data Availability

Supporting data for this manuscript are available on request from the corresponding author.

## Supplementary Materials

The following supporting information can be downloaded at: https://www.mdpi.com/article/10.3390/antibiotics14010069/s1, Table S1: Antibiogram results.
