# Supplementary material for: Monitoring the Spread of Multidrug-Resistant Escherichia coli Throughout the Broiler Production Cycle
Source: Antibiotics (Basel). 2025 Jan 10;14(1):69. doi: 10.3390/antibiotics14010069 (PMC11760847; doi:10.3390/antibiotics14010069)
Supplement: Supplementary file 1 [file antibiotics-14-00069-s001.zip › antibiotics-3396975-supplementary.pdf]

Supplementary Materials

Table S1. Antibiogram results.

STRAINS	FEP	AMC	ATM	CFZ	CTX	GEN	CLO	IPM	CIP	FOS	AMP	ENR	SUT	TET	NAL	FLF	CFO
G01_S1	S	S	S	S	S	R	S	S	R	S	R	R	R	S	R	S	S
G01_S2	S	S	S	S	S	R	S	S	R	S	S	R	R	S	R	S	S
G01_S3	S	S	S	S	S	R	S	S	R	S	S	R	R	S	R	S	S
G01_S6	S	S	S	S	S	R	S	S	R	S	R	R	S	R	R	S	S
G01_S7	S	S	S	S	S	R	S	S	R	S	R	R	R	S	R	S	S
G01_S8	S	S	S	S	S	R	S	S	R	S	S	R	R	S	R	S	S
G01_S9	S	S	S	S	S	R	S	S	R	S	R	R	S	R	R	S	S
G01_S11	S	S	S	S	S	R	S	S	R	S	R	R	R	S	R	S	S
G01_C13	S	S	S	S	S	R	S	S	R	S	R	R	R	S	R	S	S
G01_C14	S	S	S	S	S	R	S	S	R	S	R	R	R	S	R	S	S
G01_C15	S	S	S	S	S	R	S	S	R	S	R	R	R	S	R	S	S
G01_C16	S	S	S	S	S	R	S	S	R	S	R	R	R	S	R	S	S
G01_C3	S	S	S	R	S	R	S	S	R	S	R	R	S	S	R	S	S
G01_C4	S	S	S	S	S	S	S	S	R	S	S	R	S	S	R	S	S
G01_C5	S	S	R	R	R	R	S	S	R	S	R	R	S	S	R	S	S
G01_C6	S	S	S	R	S	R	S	S	R	S	R	R	S	S	R	S	S
G01_B7	S	S	S	R	S	R	S	S	R	S	R	R	S	S	R	S	S
G01_B8	S	S	S	S	S	S	S	S	R	S	S	R	S	S	R	S	S
G01_B9	R	S	S	R	R	S	S	S	R	S	R	R	R	S	R	S	S
G01_B11	S	S	S	S	S	S	S	S	R	S	S	R	S	S	R	S	S
G01_B12	S	S	S	R	S	R	S	S	R	S	R	R	R	S	R	S	S
G01_B13	S	S	S	R	S	R	S	S	R	S	R	R	R	S	R	S	S
G01_B1	S	S	S	S	S	R	S	S	S	S	S	S	S	S	S	S	S
G01_B2	R	S	R	R	R	R	S	S	S	R	R	S	S	S	S	S	S
G02_S3	S	S	S	R	S	R	S	S	S	R	S	S	S	S	S	S	S
G02_S4	R	S	R	R	R	R	S	S	S	R	R	S	S	S	S	S	S
G02_S5	S	S	S	R	S	R	S	S	S	R	S	S	S	S	S	S	S
G02_S6	S	S	S	R	S	R	S	S	R	S	R	R	R	R	R	S	S
G02_S7	S	S	S	R	S	R	S	S	R	S	R	R	R	R	R	S	S
G02_S8	S	S	S	R	S	R	S	S	R	S	R	R	R	R	R	S	S
G02_S9	S	S	S	R	S	R	S	S	S	S	S	S	S	S	S	S	S
G02_S10	R	S	R	R	R	R	S	S	S	R	R	S	S	S	S	S	S
G02_C1	S	S	S	S	S	R	S	S	S	S	S	S	S	S	S	S	S
G02_C2	S	S	S	R	S	R	S	S	R	S	R	R	R	R	R	S	S
G02_C3	R	S	R	R	R	R	S	S	S	R	R	S	S	S	S	S	S
G02_C4	S	S	S	R	S	R	S	S	R	S	R	R	R	R	R	S	S
G02_C5	S	S	S	R	S	R	R	S	S	S	R	S	S	R	S	S	S
G02_C6	S	S	S	S	S	R	S	S	S	S	S	S	S	S	S	S	S
G02_C7	R	S	R	R	R	R	S	S	S	R	R	S	S	S	S	S	S
G02_C8	S	S	S	R	S	R	S	S	R	S	R	R	R	R	R	S	S
G02_B9	R	S	R	R	R	R	S	S	S	R	R	S	S	S	S	S	S
G02_B10	S	S	S	S	S	R	S	S	S	R	S	S	S	S	S	S	S

G02_B1	R	S	S	R	R	R	S	S	R	S	R	R	S	S	R	S	S
G02_B2	S	S	S	R	S	S	S	S	S	S	S	S	R	R	S	S	S
G02_B3	R	S	R	R	R	S	R	S	R	S	R	R	R	S	R	S	S
G02_B4	S	S	S	R	R	R	S	S	S	S	S	S	S	S	S	S	S
G02_B5	S	S	S	R	R	R	S	S	S	S	S	S	S	S	S	S	S
G02_B6	S	S	S	R	R	R	S	S	S	S	S	S	S	S	S	S	S
G03_S1	R	S	R	R	R	R	S	S	S	R	R	S	S	S	S	S	S
G03_S7	R	S	R	R	R	S	R	S	R	S	R	R	R	S	R	S	S
G03_S8	R	S	R	R	R	R	S	S	S	R	R	S	S	S	S	S	S
G03_S9	R	S	R	R	R	R	S	S	S	R	R	S	S	S	S	S	S
G03_S10	R	S	R	R	R	R	S	S	S	R	R	S	S	S	S	S	S
G03_S5	R	R	S	S	S	R	S	S	S	S	R	S	S	S	S	S	R
G03_S6	S	S	S	S	S	R	S	S	S	S	S	S	S	S	R	S	S
G03_S11	S	S	S	S	S	R	S	S	R	S	R	R	R	R	R	S	S
G03_C12	S	S	S	S	S	R	S	S	R	S	S	S	S	S	R	S	S
G03_C14	S	S	S	S	S	R	S	S	R	S	R	R	R	R	R	S	S
G03_C15	S	S	S	S	S	R	S	S	R	S	S	S	S	S	R	S	S
G03_C16	S	R	S	S	S	R	S	S	R	S	R	R	R	R	R	S	S
G03_C17	S	R	S	S	S	R	S	S	S	S	R	S	S	S	S	S	R
G03_C13	S	R	S	S	S	R	S	S	S	S	R	S	S	S	S	S	R
G03_C19	S	R	S	S	S	R	S	S	S	S	R	S	S	S	S	S	R
G03_C2	S	R	S	R	S	R	S	S	S	S	R	R	S	S	R	R	R
G03_B3	R	S	R	R	R	R	S	S	R	S	R	R	S	S	R	S	S
G03_B4	S	S	S	S	S	R	S	S	S	S	S	S	S	S	S	S	S
G03_B5	S	S	S	R	S	R	S	S	S	S	R	R	S	S	R	R	R
G03_B6	S	R	S	R	S	R	S	S	S	S	R	R	S	S	R	R	R
G03_B7	R	S	R	R	R	R	S	S	R	S	R	R	S	S	R	S	S
G03_B8	S	S	S	S	S	S	S	S	S	S	S	S	S	S	R	S	S
G03_B9	S	S	S	S	S	R	S	S	S	S	S	S	S	S	S	S	S
G03_B10	S	R	S	R	S	R	S	S	S	S	R	R	S	S	R	R	R
G04_S1	R	S	R	R	R	R	S	S	R	S	R	R	S	S	R	S	S
G04_S7	S	R	S	R	S	R	S	S	R	S	R	R	S	S	R	S	S
G04_S8	R	S	R	R	R	R	S	S	R	S	R	R	S	S	R	S	S
G04_S2	S	S	S	S	S	R	S	S	S	S	S	S	S	S	S	S	S
G04_S3	S	S	S	S	S	R	S	S	S	S	S	S	S	S	S	S	S
G04_S4	S	S	S	S	S	R	S	S	S	S	S	S	S	S	S	S	S
G04_S5	S	S	S	S	S	R	S	S	S	S	S	S	S	S	S	S	S
G04_S6	S	S	S	S	S	R	S	S	S	S	S	S	S	S	S	S	S
G04_C7	S	S	S	S	S	R	S	S	S	S	S	S	S	S	S	S	S
G04_C8	S	S	S	S	S	R	S	S	R	S	S	S	S	S	S	S	S
G04_C9	S	S	S	S	S	R	S	S	S	S	S	S	S	S	S	S	S
G04_C10	S	S	S	S	S	R	S	S	S	S	S	S	S	S	S	S	S
G04_C11	R	R	R	R	R	R	S	S	R	S	R	R	S	S	R	S	S
G04_C4	S	S	S	S	S	R	S	S	R	S	R	R	R	S	R	S	S
G04_C5	R	S	R	R	R	R	S	S	R	S	R	R	R	R	R	R	S
G04_C6	S	S	S	S	S	R	S	S	R	S	R	R	S	R	R	S	S
G04_B7	S	S	S	S	S	R	S	S	R	S	R	R	R	S	R	S	S

G04_B8	R	S	R	R	R	R	S	S	R	S	R	R	R	R	R	R	S
G04_B9	S	S	S	S	S	R	S	S	R	S	R	R	R	S	R	S	S
G04_B10	S	S	S	S	S	R	S	S	R	S	S	R	S	R	R	S	S
G04_B11	R	S	R	R	R	R	S	S	R	S	R	R	R	R	R	R	S
G04_B12	S	S	S	S	S	R	S	S	R	S	R	R	R	S	R	S	S
G04_B13	R	S	R	R	R	R	S	S	R	S	R	R	R	R	R	R	S
G04_B14	S	S	S	S	S	R	S	S	R	S	R	R	R	S	R	S	S
G05_S1	S	S	S	S	S	R	R	S	R	S	R	R	R	R	R	R	S
G05_S7	S	S	S	R	R	R	S	S	R	S	R	R	S	S	R	S	S
G05_S8	S	S	S	S	S	R	S	S	R	S	S	R	S	R	R	S	S
G05_S2	S	S	S	S	S	R	S	S	R	S	S	R	S	R	R	S	S
G05_S3	S	S	S	S	S	R	R	S	R	S	R	R	R	R	R	R	S
G05_S4	R	S	R	R	R	R	S	S	R	R	R	R	R	S	R	S	S
G05_S5	S	S	S	S	S	R	R	S	R	S	R	R	R	R	R	R	S
G05_S6	S	S	S	S	S	R	S	S	R	S	S	R	S	R	R	S	S
G05_C7	S	S	S	S	S	R	S	S	R	S	S	R	S	R	R	S	S
G05_C8	S	S	S	S	S	R	R	S	R	S	R	R	R	R	R	R	S
G05_C9	S	S	S	S	S	R	S	S	R	S	S	R	S	R	R	S	S
G05_C10	S	S	S	S	S	R	S	S	R	S	S	R	S	R	R	S	S
G05_C11	S	S	S	S	S	R	R	S	R	S	R	R	R	R	R	R	S
G05_C1	R	R	R	R	R	R	S	S	R	S	R	R	S	R	R	S	S
G05_C2	S	S	S	S	S	R	R	S	R	S	R	R	R	R	R	R	S
G05_C4	R	S	R	R	R	R	S	S	R	R	R	R	R	S	R	S	S
G05_B3	S	R	S	S	S	R	R	S	S	S	R	R	R	R	R	S	S
G05_B4	S	R	S	S	S	R	R	S	S	S	R	R	R	R	R	S	S
G05_B5	R	R	S	R	R	R	S	S	S	S	R	R	S	S	R	S	S
G05_B6	S	R	S	S	S	R	R	S	S	S	R	R	R	R	R	S	S
G05_B7	R	R	S	R	R	R	S	S	S	S	R	R	S	S	R	S	S
G05_B8	S	R	S	S	S	R	R	S	S	S	R	R	R	R	R	S	S
G05_B9	S	R	S	S	S	R	R	S	S	S	R	R	R	R	R	S	S
G05_B10	S	R	S	S	S	R	R	S	S	S	R	R	R	R	R	S	S
G06_S11	S	R	S	S	S	R	R	S	S	S	R	R	R	R	R	S	S
G06_S12	S	R	S	S	S	R	R	S	S	S	R	R	R	R	R	S	S
G06_S1	R	S	R	R	R	R	S	S	S	S	R	R	R	R	R	S	S
G06_S2	S	S	S	R	S	R	S	S	R	S	S	R	R	S	R	R	S
G06_S3	R	S	R	R	R	R	S	S	S	S	R	R	R	R	R	S	S
G06_S4	R	S	R	R	R	R	S	S	S	S	R	R	R	R	R	S	S
G06_S5	S	S	S	R	S	R	S	S	R	S	S	R	R	S	R	R	S
G06_S6	S	S	S	R	S	R	S	S	R	S	S	R	R	S	R	R	S
G06_C7	S	R	R	R	R	R	S	S	S	S	R	R	R	R	R	S	S
G06_C8	S	R	R	R	R	R	S	S	S	S	R	R	R	R	R	S	S
G06_C9	S	R	R	R	R	R	S	S	S	S	R	R	R	R	R	S	S
G06_C10	S	S	S	R	S	R	S	S	R	S	S	R	R	S	R	R	S
G06_C11	S	S	S	R	S	R	S	S	R	S	R	R	R	R	R	S	S
G06_C1	S	S	S	S	S	R	S	S	R	S	S	R	R	R	R	S	S
G06_C2	S	S	S	S	S	R	S	S	R	S	S	R	R	R	R	S	S
G06_C3	S	S	S	R	S	R	S	S	R	S	R	R	R	R	R	S	S

G06_B4	S	S	S	S	S	R	S	S	R	S	S	R	R	R	R	S	S
G06_B5	S	S	S	S	S	R	S	S	R	S	S	R	R	R	R	S	S
G06_B6	S	S	S	S	S	R	S	S	R	S	S	R	R	R	R	S	S
G06_B7	S	S	S	S	S	R	S	S	R	S	S	R	R	R	R	S	S
G06_B8	S	S	S	S	S	R	S	S	R	S	S	R	R	R	R	S	S
G06_B9	S	S	S	R	S	R	S	S	R	S	R	R	R	R	R	S	S
G06_B10	S	S	S	S	S	R	S	S	R	S	S	R	R	R	R	S	S
G06_B1	R	R	S	S	S	R	S	S	R	S	R	R	R	S	R	S	S
G07_S2	R	R	S	S	S	R	S	S	R	S	R	R	R	S	R	S	S
G07_S3	R	R	R	R	R	R	S	S	S	S	R	S	R	S	R	S	S
G07_S4	R	R	R	R	R	R	S	S	S	S	R	S	R	S	R	S	S
G07_S5	R	R	S	S	S	R	S	S	R	S	R	R	R	R	R	S	S
G07_S6	S	R	S	S	S	R	S	S	R	S	R	R	R	R	R	S	S
G07_S7	R	R	R	R	R	R	S	S	S	S	R	S	R	S	R	S	S
G07_S8	R	R	S	S	S	R	S	S	R	S	R	R	R	R	R	S	S
G07_S9	R	R	S	S	S	R	S	S	R	S	R	R	R	S	R	S	S
G07_C10	S	R	S	S	S	R	S	S	R	S	R	R	R	S	R	S	S
G07_C1	S	R	S	R	S	R	S	S	R	S	R	R	R	R	R	R	S
G07_C2	S	S	S	S	S	R	S	S	R	S	R	R	R	R	R	S	S
G07_C3	S	R	S	R	S	R	S	S	R	S	R	R	R	R	R	R	S
G07_C4	R	S	R	R	R	R	S	S	R	S	R	R	R	R	R	S	S
G07_C5	S	R	S	R	S	R	S	S	R	S	R	R	R	R	R	R	S
G07_C6	R	S	R	R	R	R	S	S	R	S	R	R	R	R	R	S	S
G07_C7	S	R	S	R	S	R	S	S	R	S	R	R	R	R	R	R	S
G07_B8	R	S	R	R	R	R	S	S	R	S	R	R	R	R	R	S	S
G07_B9	S	R	S	R	S	R	S	S	R	S	R	S	R	R	R	R	S
G07_B10	R	S	R	R	R	R	S	S	R	S	R	R	R	R	R	S	S
G07_B1	S	S	S	R	S	R	S	S	R	S	R	R	R	R	R	S	S
G07_B2	S	S	S	R	S	R	S	S	R	S	R	R	R	R	R	R	S
G07_B3	S	S	S	R	S	R	S	S	R	S	R	R	R	R	R	R	S
G07_B4	S	S	S	R	S	R	S	S	R	S	R	R	R	R	R	S	S
G07_B5	S	S	S	R	S	R	S	S	R	S	R	R	R	R	R	R	S
G08_S6	S	S	S	R	S	R	S	S	R	S	R	R	R	R	R	R	S
G08_S7	S	S	S	R	S	R	S	S	R	S	R	R	R	R	R	S	S
G08_S8	S	S	S	R	S	R	S	S	R	S	R	R	R	R	R	S	S
G08_S9	S	S	S	R	S	R	S	S	R	S	R	R	R	R	R	S	S
G08_S10	R	S	R	R	R	R	S	S	R	S	R	R	R	R	R	S	S
G08_S1	S	S	S	S	S	S	R	S	R	S	R	R	R	R	R	S	S
G08_S2	S	S	S	S	S	S	S	S	R	S	R	R	R	R	R	S	S
G08_S3	S	S	S	S	S	R	S	S	R	S	R	R	R	R	R	S	S
G08_C4	S	S	S	S	S	S	R	S	R	S	R	R	R	R	R	S	S
G08_C5	S	S	S	S	S	S	S	S	S	S	R	R	R	R	R	S	S
G08_C6	S	S	S	S	S	S	R	S	R	S	R	R	R	R	R	S	S
G08_C7	S	S	S	S	S	R	S	S	R	S	R	R	R	R	R	S	S
G08_C8	S	S	S	S	S	S	R	S	R	S	R	R	R	R	R	S	S
G08_C9	S	S	S	S	S	S	R	S	R	S	R	R	R	R	R	S	S
G08_C10	S	S	S	S	S	S	R	S	R	S	R	R	R	R	R	S	S

G08_C1	S	S	S	R	S	R	R	S	R	S	R	R	R	S	R	S	S
G08_B2	S	S	S	S	S	R	S	S	R	S	R	R	S	S	R	S	S
G08_B3	S	S	S	S	S	R	S	S	R	S	R	R	S	S	R	R	S
G08_B4	S	S	S	S	S	R	S	S	R	S	S	R	S	S	R	S	S
G08_B5	S	S	S	S	S	R	S	S	R	S	R	R	S	R	S	S	S
G08_B6	S	S	S	S	S	R	R	S	R	S	S	R	R	S	R	S	S
G08_B7	S	S	S	S	S	R	S	S	R	S	R	R	S	S	R	R	S
G08_B8	S	S	S	S	S	R	S	S	R	S	R	R	S	S	R	S	S
G08_B9	S	S	S	S	S	R	S	S	R	S	R	R	S	R	S	S	S
G09_S10	S	S	S	S	S	R	R	S	R	S	S	R	R	S	R	S	S
G09_S1	S	S	S	S	S	S	S	S	R	S	S	S	S	S	R	S	S
G09_S2	S	S	S	S	S	R	S	S	R	S	S	R	S	S	R	S	S
G09_S3	S	S	S	S	S	R	S	S	R	S	S	R	S	S	R	S	S
G09_S4	S	S	S	S	S	S	S	S	R	S	S	S	S	S	R	S	S
G09_S5	S	S	S	S	S	S	S	S	R	S	S	S	S	S	R	S	S
G09_S6	S	S	S	S	S	R	S	S	R	S	S	R	S	S	R	S	S
G09_S7	S	S	S	S	S	S	S	S	R	S	S	S	S	S	R	S	S
G09_C8	S	S	S	S	S	R	S	S	R	S	S	R	S	S	R	S	S
G09_C9	S	S	S	S	S	S	S	S	R	S	S	S	S	S	R	S	S
G09_C10	S	S	S	S	S	S	S	S	R	S	S	S	S	S	R	S	S
G09_C11	S	S	S	S	S	R	S	S	R	S	S	R	S	S	R	S	S
G09_C2	R	S	R	R	R	R	S	S	R	S	R	R	S	R	R	S	S
G09_C3	S	S	S	S	S	R	S	S	R	S	R	R	S	R	R	S	S
G09_C4	S	S	S	S	S	R	S	S	R	S	R	R	S	R	R	S	S
G09_C5	R	S	R	R	R	R	S	S	R	S	R	R	S	R	R	S	S
G09_B6	S	S	S	S	S	R	S	S	R	S	R	R	S	R	R	S	S
G09_B7	R	S	R	R	R	R	S	S	R	S	R	S	S	R	R	S	S
G09_B8	S	S	S	S	S	R	S	S	R	S	R	R	S	R	R	S	S
G09_B9	S	S	S	S	S	R	S	S	R	S	R	R	S	R	R	S	S
G09_B10	R	S	R	R	R	R	S	S	R	S	R	R	S	R	R	S	S
G09_B11	S	S	S	S	S	R	S	S	R	S	R	R	S	R	R	S	S
G09_B12	S	S	S	S	S	R	S	S	R	S	R	R	S	R	R	S	S
G09_B13	R	S	R	R	R	R	S	S	R	S	R	R	R	R	R	S	S
G010_S1	S	S	S	S	S	S	S	S	S	S	S	S	S	S	R	S	S
G010_S2	S	S	S	S	S	R	S	S	R	S	R	R	R	R	R	S	S
G010_S3	S	S	S	S	S	R	S	S	R	S	R	R	R	R	R	S	S
G010_S4	S	S	S	S	S	R	S	S	R	S	R	R	R	R	R	S	S
G010_S5	S	S	S	S	S	S	S	S	S	S	S	S	S	S	R	S	S
G010_S6	S	S	S	S	S	S	S	S	S	S	S	S	S	S	R	S	S
G010_S7	S	S	S	R	S	R	S	S	R	S	R	R	R	R	R	S	S
G010_S8	R	S	R	R	R	R	R	S	R	S	R	R	R	R	R	R	S
G010_C9	S	S	S	S	S	S	S	S	S	S	S	S	S	S	R	S	S
G010_C10	S	S	S	S	S	R	S	S	R	S	R	R	R	R	R	S	S
G010_C1	S	S	S	R	S	R	S	S	S	S	R	S	S	S	S	S	S
G010_C2	S	S	S	S	S	R	S	S	S	S	R	S	S	S	S	S	S
G010_C3	S	S	S	S	S	R	S	S	S	S	R	S	S	R	S	S	S
G010_C4	S	S	S	S	S	R	S	S	S	S	R	S	S	S	S	S	S

G010_C5	R	S	S	R	R	R	R	S	R	S	R	R	R	R	R	R	S
G010_C6	S	S	S	S	S	R	S	S	S	S	R	S	S	S	S	S	S
G010_B7	S	S	S	S	S	R	S	S	S	S	R	S	S	S	S	S	S
G010_B8	R	S	S	R	R	R	R	S	R	S	R	R	R	R	R	R	S
G010_B9	S	S	S	S	S	R	S	S	S	S	R	S	S	S	S	S	S
G010_B10	S	S	S	S	S	R	S	S	S	S	R	S	S	S	S	S	S
G010_B2	S	S	S	R	S	S	S	S	R	S	S	R	S	S	R	S	S
G010_B3	S	S	S	R	S	R	S	S	R	S	S	R	R	S	R	S	S
G010_B4	R	S	R	R	R	R	S	S	R	R	R	R	R	S	R	S	S
G010_B5	S	S	S	R	S	R	S	S	R	S	S	R	R	S	R	S	S
G011_S6	S	S	S	R	S	R	S	S	R	S	S	R	R	S	R	S	S
G011_S7	S	S	S	R	S	S	S	S	R	S	S	R	S	S	R	S	S
G011_S8	S	S	S	R	S	R	S	S	R	S	S	R	R	S	R	S	S
G011_S9	S	S	S	S	S	R	S	S	R	S	S	R	R	S	R	S	S
G011_S10	S	S	S	R	S	S	S	S	R	S	S	R	S	S	R	S	S
G011_S11	S	S	S	S	S	S	S	S	R	S	S	R	S	S	R	S	S
G011_S2	S	S	S	R	S	S	S	S	R	S	R	R	S	R	R	S	S
G011_S3	R	S	R	R	R	S	S	S	R	S	R	S	S	R	R	S	S
G011_C4	S	S	S	R	S	S	S	S	R	S	R	R	S	R	R	S	S
G011_C5	S	S	S	R	S	S	S	S	R	S	R	R	S	R	R	S	S
G011_C6	R	S	R	R	R	S	S	S	R	S	R	R	S	R	R	S	S
G011_C7	S	S	S	R	S	S	S	S	R	S	R	R	S	R	R	S	S
G011_C8	S	S	S	R	S	S	S	S	R	S	R	R	S	R	R	S	S
G011_C9	R	S	R	R	R	S	S	S	R	S	R	R	S	R	R	S	S
G011_C10	R	S	R	R	R	S	S	S	R	S	R	R	S	R	R	S	S
G011_C11	R	S	R	R	R	S	S	S	R	S	R	R	S	R	R	S	S
G011_B1	R	S	R	R	R	R	R	S	S	R	R	R	R	R	R	R	R
G011_B2	S	S	S	S	S	S	S	S	S	S	R	R	S	R	R	S	S
G011_B3	S	S	S	S	S	S	S	S	S	S	R	R	S	R	R	S	S
G011_B4	R	S	R	R	R	R	R	S	S	R	R	R	R	R	R	R	R
G011_B5	S	S	S	S	S	S	S	S	S	S	R	R	S	R	R	S	S
G011_B6	S	S	S	S	S	S	S	S	S	S	R	R	S	R	R	S	S
G011_B7	R	S	R	R	R	R	R	S	S	R	R	R	R	R	R	R	R
G011_B8	S	S	S	S	S	S	S	S	S	S	R	R	S	R	R	S	S
G012_S9	S	S	S	S	S	S	S	S	S	S	R	R	S	R	R	S	S
G012_S10	R	S	R	R	R	R	R	S	S	R	R	R	R	R	R	R	R
G012_S1	R	S	S	R	R	R	S	S	R	S	R	R	S	R	R	S	S
G012_S2	S	S	S	S	S	R	S	S	R	S	S	R	S	S	R	S	S
G012_S3	S	S	S	S	S	R	S	S	R	S	S	R	S	S	R	S	S
G012_S4	R	S	S	R	R	R	S	S	R	S	R	R	S	R	R	S	S
G012_S5	R	S	S	R	R	R	S	S	R	S	R	R	S	R	R	S	S
G012_S6	S	S	S	S	S	R	S	S	R	S	S	R	S	S	R	S	S
G012_C7	S	S	S	S	S	R	S	S	R	S	S	R	S	S	R	S	S
G012_C8	R	S	S	R	R	R	S	S	R	S	R	R	S	R	R	S	S
G012_C9	S	S	S	S	S	R	S	S	R	S	S	R	S	S	R	S	S
G012_C10	R	S	S	R	R	R	S	S	R	S	R	R	S	R	R	S	S
G012_C1	R	S	S	R	R	R	S	S	R	S	R	R	S	R	R	S	S

G012_C2	R	S	S	R	R	R	S	S	R	S	R	R	S	R	R	S	S
G012_C3	S	S	S	S	S	R	S	S	R	S	S	R	S	S	R	S	S
G012_C4	R	S	S	R	R	R	S	S	R	S	R	R	S	R	R	S	S
G012_B5	R	S	S	R	R	R	S	S	R	S	R	R	S	R	R	S	S
G012_B6	S	S	S	S	S	R	S	S	R	S	S	R	S	S	R	S	S
G012_B7	S	S	S	S	S	R	S	S	R	S	S	R	S	S	R	S	S
G012_B8	R	S	S	R	R	R	S	S	R	S	R	R	S	R	R	S	S
G012_B9	S	S	S	S	S	R	S	S	R	S	S	R	S	S	R	S	S
G012_B1	R	S	S	R	R	R	S	S	R	S	R	R	S	R	R	S	S
G012_B2	R	S	R	R	R	R	S	S	S	S	R	R	S	R	R	S	S
G012_B3	S	S	S	R	S	R	S	S	R	S	S	R	S	S	R	S	S
G013_S4	R	S	R	R	R	R	S	S	R	S	R	R	S	S	R	S	S
G013_S5	R	S	R	R	R	R	S	S	S	S	R	R	S	R	R	S	S
G013_S6	R	S	R	R	R	R	S	S	R	S	R	R	S	S	R	S	S
G013_S7	R	S	R	R	R	R	S	S	R	S	R	R	S	S	R	S	S
G013_S8	R	S	R	R	R	R	S	S	S	S	R	R	S	R	R	S	S
G013_S9	R	S	R	R	R	R	S	S	S	S	R	R	S	R	R	S	S
G013_S10	R	S	R	R	R	R	S	S	R	S	R	R	S	S	R	S	S
G013_S11	R	S	R	R	R	R	S	S	R	S	R	R	S	S	R	S	S
G013_C1	R	R	R	R	R	R	R	S	S	S	R	S	R	R	s	S	S
G013_C2	S	R	S	S	S	S	S	S	S	S	R	S	S	R	s	S	S
G013_C3	R	S	R	R	R	R	R	S	S	S	R	S	R	R	s	S	S
G013_C4	S	S	S	S	S	S	S	S	S	S	R	S	S	R	S	S	S
G013_C5	R	S	R	R	R	R	R	S	S	S	R	R	R	R	S	S	S
G013_C6	S	S	S	S	S	S	S	S	S	S	R	S	S	R	S	S	S
G013_C7	S	S	S	S	S	S	S	S	S	S	R	R	S	R	S	S	S
G013_C8	R	S	R	R	R	S	S	S	S	S	R	S	S	S	S	S	S
G013_B9	R	S	R	R	R	S	S	S	S	S	R	S	S	S	S	S	S
G013_B10	S	S	S	S	S	S	S	S	S	S	R	S	S	R	R	S	S
G013_B1	R	S	S	S	S	R	S	S	S	S	R	S	S	S	S	S	R
G013_B2	R	S	R	R	R	S	S	S	S	S	R	S	S	S	S	S	S
G013_B3	R	S	R	R	R	R	R	S	S	S	R	R	R	R	R	S	S
G013_B4	S	S	S	R	S	S	S	S	S	S	R	S	S	S	R	S	S
G013_B5	R	S	R	R	R	R	S	S	R	S	R	R	S	S	R	S	S
G013_B6	S	S	S	S	S	S	S	S	S	S	R	R	S	R	S	S	S
G014_S7	S	S	S	R	S	S	S	S	S	S	R	R	S	S	R	S	S
G014_S8	R	S	R	R	R	R	S	S	R	S	R	R	S	S	R	S	S
G014_S9	R	S	R	R	R	R	S	S	R	S	R	R	S	S	R	S	S
G014_S10	S	S	S	R	S	S	S	S	S	S	R	R	S	S	R	S	S
G014_S1	S	S	S	R	S	S	S	S	S	S	R	S	S	S	R	S	S
G014_S2	S	S	S	S	S	S	S	S	S	S	S	S	S	S	S	S	S
G014_S3	S	S	S	R	S	S	S	S	S	S	R	S	S	S	R	S	S
G014_S4	S	S	S	S	S	S	S	S	S	S	S	S	S	S	S	S	S
G014_C5	S	S	S	S	S	S	R	S	R	S	R	S	S	S	R	S	S
G014_C6	S	S	S	R	S	R	S	S	S	S	R	S	S	S	R	S	R
G014_C7	S	S	S	R	S	S	S	S	S	S	R	S	S	S	R	S	S
G014_C8	S	S	S	R	S	S	S	S	S	S	R	S	S	S	R	S	S

G014_C9	S	S	S	R	S	S	S	S	S	S	R	S	S	S	R	S	S
G014_C10	S	S	S	R	S	S	S	S	R	S	R	S	S	S	R	S	S
G014_C11	S	S	S	R	S	R	S	S	S	S	R	S	S	S	R	S	R
G014_C1	R	S	R	R	R	S	R	S	R	S	R	R	S	S	R	S	S
G014_B2	R	S	R	R	R	S	R	S	R	S	R	R	S	S	R	S	S
G014_B3	R	S	R	R	R	S	R	S	R	S	R	R	S	S	R	S	S
G014_B4	S	S	S	S	S	R	R	S	R	S	R	R	R	S	R	S	S
G014_B5	R	S	R	R	R	S	R	S	R	S	R	R	S	S	R	S	S
G014_B6	S	S	S	S	S	R	R	S	R	S	R	R	R	S	R	S	S
G014_B7	R	S	R	R	R	S	R	S	R	S	R	R	S	S	R	S	S
G014_B8	S	S	S	S	S	R	R	S	R	S	R	R	R	S	R	S	S
G014_B9	R	S	R	R	R	S	R	S	R	S	R	R	S	S	R	S	S
G015_S10	S	S	S	S	S	R	R	S	R	S	R	R	R	S	R	S	S
G015_S11	R	S	R	R	R	S	R	S	R	S	R	R	S	S	R	S	S
G015_S1	S	S	S	S	S	R	R	S	R	S	R	R	R	S	R	S	S
G015_S2	S	S	S	R	R	R	S	S	R	S	R	R	R	R	R	S	S
G015_S3	S	S	S	S	S	R	R	S	R	S	R	R	R	S	R	S	S
G015_S4	S	S	S	S	S	R	R	S	R	S	R	R	R	S	R	S	S
G015_S5	R	S	S	R	R	R	S	S	R	S	R	R	R	R	R	S	S
G015_S6	S	S	S	S	S	R	S	S	R	S	R	R	R	S	R	S	S
G015_C7	S	S	S	S	S	R	S	S	R	S	R	R	R	S	R	S	S
G015_C8	S	S	S	S	S	R	R	S	R	S	R	R	R	S	R	S	S
G015_C9	R	S	S	R	R	R	S	S	R	S	R	R	R	R	R	S	S
G015_C10	S	S	S	S	S	R	R	S	R	S	R	R	R	S	R	S	S
G015_C11	R	S	S	R	R	R	S	S	R	S	R	R	R	R	R	S	S
G015_C4	S	S	S	S	S	S	S	S	R	S	R	R	R	S	R	S	S
G015_C5	S	S	S	S	S	S	S	S	R	S	R	R	R	S	R	S	S
G015_C6	S	S	S	S	S	S	S	S	R	S	R	R	R	S	R	S	S
G015_B7	S	S	S	S	S	S	S	S	R	S	R	R	R	S	R	S	S
G015_B8	S	S	S	S	S	S	S	S	R	S	R	R	R	S	R	S	S
G015_B9	S	S	S	S	S	S	S	S	R	S	R	R	R	S	R	S	S
G015_B10	S	S	S	S	S	S	S	S	R	S	R	R	R	S	R	S	S
G015_B11	S	S	S	S	S	S	S	S	R	S	R	R	R	S	R	S	S
G015_B12	S	S	S	S	S	S	S	S	R	S	R	R	R	S	R	S	S
G015_B13	S	S	S	S	S	S	S	S	R	S	R	R	R	S	R	S	S
G015_B1	S	S	S	S	S	R	S	S	R	S	S	S	S	R	R	S	S
G016_S2	S	S	S	R	R	R	S	S	R	S	R	R	R	R	R	S	R
G016_S3	S	S	S	R	R	R	S	S	R	S	R	R	R	R	R	S	R
G016_S4	S	S	S	S	S	R	S	S	R	S	S	S	S	R	R	S	S
G016_S5	S	S	S	R	R	R	S	S	R	S	R	R	R	R	R	S	R
G016_S6	S	S	S	S	S	R	S	S	R	S	S	S	S	R	R	S	S
G016_S7	S	S	S	S	S	R	S	S	R	S	S	S	S	R	R	S	S
G016_S8	R	S	R	R	R	R	S	S	R	S	R	R	S	R	R	S	S
G016_S9	S	S	S	S	S	R	S	S	R	S	S	S	S	R	R	S	S
G016_C10	S	S	S	R	R	R	S	S	R	S	R	R	R	R	R	S	R
G016_C6	S	S	S	S	S	S	S	S	S	S	S	S	S	S	R	S	S
G016_C7	R	S	R	S	R	S	S	S	R	R	S	S	S	S	R	S	R

G016_C8	R	S	R	R	R	R	S	S	R	S	R	R	S	R	R	S	S
G016_C9	R	S	R	S	R	S	S	S	R	R	S	S	S	S	R	S	R
G016_C10	R	S	R	S	R	S	S	S	R	R	S	S	R	S	R	S	R
G016_C11	S	S	S	S	S	R	S	S	S	S	S	S	S	R	R	S	S
G016_C12	S	S	S	S	S	S	S	S	S	S	S	S	S	S	R	S	S
G016_B13	S	S	S	S	S	S	R	S	S	S	R	S	S	S	R	R	S
G016_B14	S	S	S	S	S	R	S	S	S	S	S	S	S	S	R	S	S
G016_B15	S	S	S	S	S	S	S	S	S	S	S	S	S	S	R	S	S
G016_B3	S	S	S	S	S	S	R	S	S	S	R	S	R	S	R	R	S
G016_B4	S	S	S	S	S	S	R	S	S	S	R	S	R	S	R	R	S
G016_B5	S	S	S	R	R	S	R	S	R	S	R	R	R	R	R	R	S
G016_B6	S	S	S	S	S	S	R	S	S	S	R	S	R	S	R	R	S
G016_B7	S	S	S	R	R	S	R	S	R	S	R	R	R	R	R	R	S
G017_S8	S	S	S	S	S	S	R	S	S	S	R	S	R	S	R	R	S
G017_S9	S	S	S	R	R	S	R	S	R	S	R	R	R	R	R	R	S
G017_S10	S	S	S	S	S	S	R	S	S	S	R	S	R	S	R	R	S
G017_S11	S	S	S	S	S	S	R	S	S	S	R	S	R	S	R	R	S
G017_S12	S	S	S	S	S	S	R	S	S	S	R	S	R	S	R	R	S
G017_ S1	R	S	R	R	R	R	R	S	R	R	R	R	R	R	R	S	R
G017_ S2	S	R	S	R	S	S	S	S	R	S	R	R	R	R	R	R	S
G017_ S3	R	S	R	R	R	R	R	S	R	S	R	R	S	R	R	R	S
G017_ C4	R	S	R	R	R	R	R	S	R	S	R	R	S	R	R	R	S
G017_ C5	R	S	R	R	R	S	R	S	R	S	R	R	R	R	R	S	S
G017_ C6	R	S	R	R	R	R	R	S	R	S	R	R	R	R	R	S	R
G017_ C7	R	S	R	R	R	R	R	S	R	S	R	R	S	R	R	R	S
G017_ C8	R	S	S	R	S	R	S	S	R	S	R	R	R	R	R	R	S
G017_ C9	S	S	R	R	R	R	R	S	R	S	R	R	R	R	R	R	S
G017_C10	R	S	R	R	R	R	R	S	R	S	R	R	S	R	R	R	S
G017_ C1	R	S	S	R	S	S	S	S	R	S	R	R	R	R	R	R	R
G017_ B2	S	S	R	R	R	S	R	S	R	R	R	R	S	S	R	S	S
G017_ B3	S	S	R	R	R	R	R	S	R	S	R	R	R	R	R	R	S
G017_ B4	R	S	R	R	R	R	R	S	R	S	R	R	S	R	R	R	S
G017_ B5	R	S	S	R	S	R	S	S	R	S	R	R	R	R	R	R	S
G017_ B6	R	S	R	R	R	R	R	S	R	S	R	R	S	R	R	R	S
G017_ B7	S	S	R	R	R	R	R	S	R	S	R	R	R	R	R	R	S
G017_ B8	R	R	S	R	S	R	S	S	R	S	R	R	R	R	R	R	S
G017_ B9	R	R	R	R	R	R	R	S	R	S	R	R	S	R	R	R	S
G018_ S1	S	S	R	R	R	R	R	S	R	S	R	R	R	R	R	R	S
G018_ S2	R	S	R	R	R	S	R	S	R	S	R	R	R	R	R	R	S
G018_S3	R	S	R	R	R	S	R	S	R	S	R	R	R	R	R	R	S
G018_ S4	S	S	S	S	S	S	S	S	R	S	R	R	S	S	R	S	S
G018_ S5	S	S	S	S	S	S	S	S	R	S	R	R	S	S	R	S	S
G018_ S8	R	S	R	R	R	S	R	S	R	S	R	R	R	S	R	R	S
G018_ S9	R	S	R	R	R	S	R	S	R	S	R	R	R	R	R	S	S
G018_ S10	R	S	R	R	R	R	R	S	R	S	R	R	R	S	R	S	S
G018_ C6	S	S	S	S	S	S	S	S	R	S	R	R	S	S	R	S	S
G018_ C7	R	S	R	R	R	S	R	S	R	S	R	R	R	S	R	R	S

G018_ C8	S	S	S	S	S	S	S	S	R	S	R	R	S	S	R	S	S
G018_ C9	S	S	S	S	S	S	S	S	R	S	R	R	S	S	R	S	S
G018_ C10	R	S	R	R	R	S	R	S	R	S	R	R	R	S	R	R	S
G018_ C4	R	S	R	R	R	R	R	S	R	S	R	R	R	R	R	S	S
G018_ C5	R	S	S	R	R	S	R	S	R	S	R	R	R	R	R	S	S
G018_ C6	S	S	S	S	S	S	S	S	R	S	R	R	S	S	R	S	S
G018_ B7	S	S	S	R	S	S	S	S	R	S	R	R	S	S	R	S	S
G018_ B8	S	S	S	R	S	S	S	S	R	S	R	R	S	S	R	S	S
G018_ B9	S	S	S	S	S	S	S	S	R	S	R	R	S	S	R	S	S
G018_ B10	S	S	S	S	S	S	S	S	R	S	R	R	S	S	R	S	S
G018_ B11	S	S	S	R	S	S	S	S	R	S	R	R	S	S	R	S	S
G018_ B12	S	S	S	S	S	S	S	S	R	S	R	R	S	S	R	S	S
G018_ B13	S	S	S	S	S	S	S	S	R	S	R	R	S	S	R	S	S
G018_ B3	S	S	R	R	S	S	S	S	R	S	R	R	S	S	R	R	S
G019_ S4	R	S	S	R	R	R	R	S	R	R	R	R	R	R	R	S	S
G019_ S5	S	S	S	S	S	S	S	S	R	S	R	R	S	S	R	S	S
G019_ S6	R	S	S	R	R	S	R	S	R	S	R	R	S	S	R	S	S
G019_ S7	S	S	S	R	S	S	S	S	R	S	R	R	R	R	R	S	S
G019_ S8	R	R	R	R	R	S	R	S	R	S	R	R	R	R	R	S	S
G019_ S9	S	S	S	S	S	S	S	S	R	S	R	R	S	S	R	S	S
G019_ S10	R	S	S	R	R	S	R	S	R	S	R	R	S	S	R	S	S
G019_ S11	S	S	S	S	S	S	S	S	R	S	R	R	S	S	R	S	S
G019_ C12	R	R	R	R	R	S	R	S	R	S	R	R	R	R	R	S	S
G019_C1	S	S	S	R	S	R	S	S	R	S	R	R	R	R	R	S	S
G019_C2	S	S	S	R	S	R	S	S	R	S	R	R	R	R	R	S	S
G019_C3	R	S	R	R	R	S	R	S	R	R	R	R	R	R	R	R	S
G019_C4	R	S	R	R	R	S	R	S	R	R	R	R	R	R	R	R	S
G019_C5	R	S	S	R	S	R	S	S	R	S	R	R	R	R	R	S	S
G019_C6	S	S	S	R	S	R	S	S	R	S	R	R	R	R	R	S	S
G019_C7	R	S	R	R	R	S	R	S	R	R	R	R	R	R	R	R	S
G019_B8	R	S	R	R	R	S	R	S	R	R	R	R	R	R	R	R	S
G019_B9	S	S	S	R	S	R	S	S	R	S	R	R	R	R	R	S	S
G019_AB10	S	S	S	R	S	R	S	S	R	S	R	R	R	R	R	S	S
G019_B11	S	S	S	R	S	R	S	S	R	S	R	R	R	R	R	S	S
G019_B12	S	S	S	R	S	R	S	S	R	S	R	R	R	R	R	S	S
G019_B13	R	S	R	R	R	S	R	S	R	R	R	R	S	S	R	S	S
G019_B14	R	S	R	R	R	S	R	S	R	R	R	R	R	R	R	R	S
G019_B16	S	S	S	R	S	R	S	S	R	S	R	R	R	R	R	S	S
G020_S1	R	S	R	R	R	S	R	S	R	R	R	R	S	S	R	S	S
G020_S2	S	S	S	R	S	R	S	S	R	S	R	R	R	R	R	S	S
G020_S3	R	S	R	R	R	S	R	S	R	R	R	R	S	S	R	S	S
G020_S4	R	S	R	R	R	S	R	S	R	R	R	R	S	S	R	S	S
G020_S5	R	S	R	R	R	S	R	S	R	S	R	R	S	R	R	S	S
G020_S6	R	S	R	R	R	R	R	S	R	R	R	R	R	S	R	S	S
G020_S8	R	S	R	R	R	S	R	S	R	S	R	R	S	R	R	S	S
G020_S9	S	S	S	R	R	R	R	S	R	S	R	R	R	S	R	R	S
G020_C6	R	S	R	R	R	S	R	S	R	S	R	R	S	R	R	S	S

G020_C7	S	S	S	R	R	R	R	S	R	S	R	R	R	S	R	R	S
G020_C8	R	S	R	R	R	S	R	S	R	R	R	R	R	R	R	R	S
G020_C9	S	S	S	R	S	R	S	S	R	S	R	R	R	R	R	S	S
G020_C10	R	S	R	R	R	S	R	S	R	S	R	R	S	R	R	S	S
G020_ C1	R	S	S	R	R	S	R	S	R	S	R	R	R	S	R	S	R
G020_ C2	R	S	R	R	R	R	R	S	R	R	R	R	R	R	R	R	S
G020_ C3	R	S	R	R	R	R	R	S	R	S	R	R	R	R	R	S	S
G020_ B4	R	S	S	R	R	R	R	S	R	R	R	R	S	R	R	S	S
G020_ B5	S	S	R	R	R	R	S	S	R	S	R	R	R	R	R	S	S
G020_ B1	R	S	R	R	R	R	R	S	R	R	R	R	S	R	R	S	S
G020_ B2	R	S	S	R	R	S	R	S	R	R	R	R	S	S	R	S	S
G020_ B3	R	S	R	R	R	S	R	S	R	S	R	R	R	S	R	S	S
G020_ B4	R	S	S	R	R	R	R	S	R	S	R	R	R	R	R	S	S
G020_ B5	R	S	S	R	R	R	R	S	R	S	R	R	R	R	R	S	S
G020_ B1	R	S	R	R	R	S	R	S	R	S	R	R	R	S	R	S	R
G021_ S2	R	R	R	R	R	S	R	S	R	S	R	R	R	S	R	S	S
G021_ S3	R	S	R	R	R	S	R	S	R	S	R	R	R	S	R	S	R
G021_ S4	R	R	S	R	R	R	R	S	R	S	R	R	R	S	R	S	S
G021_ S5	R	S	R	R	R	R	R	S	R	S	R	R	R	S	R	S	R
G021_S2	S	S	S	R	S	S	S	S	R	S	R	R	R	S	R	S	S
G021_S3	S	S	S	S	S	S	S	S	R	S	R	R	R	S	R	S	S
G021_S4	S	S	S	S	S	S	S	S	R	S	R	R	R	S	R	S	S
G021_S5	R	S	R	R	R	R	R	S	R	S	R	R	S	R	R	S	S
G021_C1	S	S	S	S	S	R	R	S	R	S	R	R	R	S	R	S	R
G021_C2	S	S	S	S	S	S	S	S	R	S	S	R	S	S	R	S	S
G021_C3	S	R	S	R	S	R	S	S	R	S	R	R	R	R	R	S	R
G021_C4	R	S	S	R	R	S	R	S	R	R	R	R	R	S	R	S	S
G021_C5	R	S	R	R	R	S	S	S	R	R	R	R	S	S	R	S	S
G021_C1	R	S	S	R	R	R	S	S	R	R	R	R	R	S	R	S	S
G021_C2	S	S	S	S	S	S	S	S	S	S	R	S	S	S	R	S	S
G021_C3	S	S	S	R	R	S	S	S	S	R	R	R	R	S	R	S	S
G021_B4	S	S	S	R	R	R	R	S	R	S	R	R	R	R	R	R	R
G021_B5	S	S	S	S	S	S	S	S	S	S	R	R	R	S	R	S	S
G021_B1	S	S	S	S	S	S	S	S	R	S	R	R	S	R	R	R	R
G021_B2	S	S	S	S	S	S	S	S	R	S	R	R	S	R	R	S	R
G021_B3	S	R	S	S	S	S	S	S	R	S	R	R	S	R	R	S	R
G021_B4	S	R	S	S	S	S	S	S	R	S	R	R	S	R	R	S	R
G021_B5	S	S	S	S	S	S	S	S	R	S	R	R	S	R	R	S	R
G021_B6	S	S	S	S	S	S	S	S	R	S	R	R	S	R	R	S	R
G022_S7	S	R	S	S	S	S	S	S	R	S	R	R	S	R	R	S	R
G022_S8	S	S	S	S	S	S	S	S	R	S	R	R	S	R	R	S	R
G022_S9	S	S	S	S	S	S	S	S	R	S	R	R	S	R	R	S	R
G022_S10	S	S	S	S	S	S	S	S	R	S	R	R	S	R	R	S	R
G022_S1	S	R	S	R	R	R	R	S	R	S	R	R	S	S	R	R	S
G022_S2	S	R	S	R	S	R	R	S	R	S	R	R	S	S	R	R	S
G022_S3	S	R	S	R	S	R	R	S	R	S	R	R	S	S	R	R	S
G022_S4	S	R	S	R	R	R	R	S	R	S	R	S	S	S	R	R	S

G022_C5	S	S	S	R	S	R	R	S	R	S	R	R	S	S	R	R	S
G022_C6	S	S	S	S	S	S	S	S	R	S	R	R	S	R	R	S	R
G022_C7	S	R	S	R	R	R	R	S	R	S	R	R	S	S	R	R	S
G022_C8	S	S	R	R	R	R	R	S	R	S	R	R	R	R	R	R	S
G022_C9	S	R	S	R	S	R	R	S	R	S	R	R	S	S	R	R	S
G022_C10	S	S	S	S	S	S	S	S	R	S	R	R	S	R	R	S	R
G022_C1	R	S	S	R	R	R	R	S	R	S	R	R	R	R	R	R	S
G022_C2	R	R	S	R	R	R	R	S	R	S	R	S	R	R	R	R	S
G022_B3	R	S	R	R	R	R	R	S	R	S	R	R	S	R	R	R	S
G022_B4	R	R	R	R	R	R	R	S	R	S	R	R	S	S	R	R	R
G022_B5	R	R	R	R	R	R	R	S	R	S	R	R	S	S	R	R	R
G022_B6	S	S	S	S	S	S	S	S	R	S	R	R	S	R	R	S	R
G022_B7	S	S	S	S	S	S	S	S	R	S	R	R	S	R	R	S	R
G022_B8	S	S	S	S	S	S	S	S	R	S	R	R	S	R	R	S	R
G022_B9	R	S	R	R	R	R	R	S	R	S	R	R	R	R	R	R	S
G022_B2	R	R	R	R	R	S	S	S	R	S	R	R	S	S	R	S	S
G023_S3	S	S	S	R	S	S	S	S	R	S	S	R	S	S	R	S	R
G023_S4	S	S	S	R	S	S	S	S	R	S	S	R	S	S	R	S	R
G023_S5	S	S	S	R	S	S	S	S	R	S	S	R	S	S	R	S	R
G023_S6	R	S	R	R	R	R	S	S	R	R	R	R	S	S	R	S	S
G023_S7	S	S	S	R	S	S	S	S	R	S	S	R	S	S	R	S	R
G023_S8	S	S	S	R	S	S	S	S	R	S	S	R	S	S	R	S	R
G023_S9	R	R	R	R	R	S	S	S	R	S	R	R	S	S	R	S	S
G023_S10	S	S	S	R	S	S	S	S	R	S	S	R	S	S	R	S	R
G023_C11	R	S	R	R	R	R	S	S	R	R	R	R	S	S	R	S	S
G023_C2	R	R	R	R	S	R	S	S	R	S	R	R	R	S	R	S	S
G023_C3	R	S	R	R	R	S	S	S	R	S	R	R	S	S	R	S	S
G023_C4	R	S	R	R	S	R	S	S	R	S	R	R	R	S	R	S	S
G023_C5	R	S	R	R	R	S	S	S	R	S	R	R	S	S	R	S	S
G023_C6	S	S	S	R	S	S	S	S	R	S	S	R	S	S	R	S	R
G023_C7	R	R	R	R	S	R	S	S	R	S	R	R	R	S	R	S	S
G023_C8	R	S	S	R	S	S	S	S	R	S	S	R	S	S	R	S	R
G023_B9	R	S	R	R	R	R	S	S	R	R	R	R	S	S	R	S	S
G023_B10	R	S	R	R	R	R	S	S	R	R	R	R	S	S	R	S	S
G023_B11	S	S	S	R	S	S	S	S	R	S	S	R	S	S	R	S	R
G023_B12	R	S	R	R	R	R	S	S	R	R	R	R	S	S	R	S	S
G023_B1	R	S	R	R	R	R	S	S	R	S	R	R	R	S	R	S	S
G023_B2	R	S	R	R	S	R	S	S	R	S	R	R	R	S	R	S	S
G023_B3	R	S	R	R	R	R	S	S	R	S	R	R	R	S	R	S	R
G023_B4	S	S	S	R	R	R	S	S	R	R	R	R	S	S	R	S	S
G024_S5	R	S	R	R	S	S	S	S	R	S	R	R	S	S	R	S	R
G024_S6	R	S	R	R	R	R	S	S	R	R	R	R	S	S	R	S	S
G024_S7	R	S	R	R	R	R	S	S	R	S	R	R	R	S	R	S	S
G024_S8	R	S	R	R	R	R	S	S	R	R	R	R	S	S	R	S	S
G024_S9	R	S	R	R	R	R	S	S	R	S	R	R	R	S	R	S	S
G024_S10	R	S	R	R	R	R	S	S	R	R	R	R	S	S	R	S	S
G024_S1	S	R	S	R	S	S	S	S	R	S	S	R	S	S	R	S	S

G024_S2	R	S	R	R	R	S	S	S	R	S	R	R	S	S	R	S	S
G024_C3	R	S	R	R	R	R	S	S	R	S	R	R	R	R	R	S	S
G024_C4	R	S	S	R	R	S	S	S	R	S	R	R	S	S	R	S	S
G024_C5	R	S	R	R	R	S	S	S	R	S	R	R	R	S	R	S	S
G024_C6	R	S	R	R	R	S	S	S	R	S	R	R	R	S	R	S	S
G024_C7	R	S	R	R	R	S	S	S	R	S	R	R	S	S	R	S	S
G024_C8	S	R	S	R	S	S	S	S	R	S	S	R	S	S	R	S	S
G024_C9	S	S	S	R	S	S	S	S	R	S	S	R	S	S	R	S	S
G024_C10	S	R	S	R	S	S	S	S	R	S	S	R	S	S	R	S	S
G024_B1	R	S	S	R	R	S	S	S	R	S	R	R	R	R	R	S	R
G024_B2	R	S	R	R	R	S	S	S	R	S	R	R	S	S	R	S	R
G024_B3	R	S	R	R	R	S	R	S	R	S	R	R	S	S	R	S	R
G024_B4	R	S	R	R	R	S	S	S	R	R	R	R	R	S	R	S	R
G024_B5	R	S	S	R	R	R	S	S	R	S	R	R	S	S	R	S	R
G024_B6	R	S	R	R	R	R	S	S	R	R	R	R	R	S	R	S	R
G024_B7	S	S	R	R	S	R	S	S	R	S	R	R	R	S	R	S	R
G024_B8	R	S	S	R	R	R	S	S	R	S	R	R	S	S	R	S	R
G025_S9	R	S	R	R	R	S	S	S	R	R	R	R	R	S	R	S	R
G025_S10	R	S	S	R	R	R	S	S	R	S	R	R	S	S	R	S	R
G025_S3	R	S	R	R	R	R	R	S	R	R	R	R	R	R	R	R	S
G025_S4	R	S	R	R	R	S	S	S	R	S	R	R	S	S	R	S	S
G025_S5	R	S	R	R	R	S	R	S	R	S	R	R	R	S	R	R	S
G025_S6	R	S	R	R	R	S	R	S	R	S	R	R	R	S	R	R	S
G025_S7	R	S	R	R	R	S	R	S	R	S	R	R	R	S	R	R	S
G025_S1	R	S	S	R	R	S	S	S	R	S	R	R	S	S	S	S	S
G025_C2	R	S	R	R	R	S	S	S	R	S	R	R	S	R	S	S	S
G025_C3	R	S	R	R	R	S	S	S	R	S	R	R	R	S	S	S	S
G025_C4	R	S	R	R	R	R	R	S	R	R	R	R	R	R	R	S	S
G025_C5	R	S	R	R	R	R	R	S	R	R	R	R	R	R	R	S	S
G025_C6	R	S	R	R	R	R	R	S	R	R	R	R	R	R	R	R	S
G025_C7	R	S	R	R	R	R	R	S	R	R	R	R	R	R	R	R	S
G025_C2	R	S	R	R	R	R	R	S	R	S	R	R	R	S	R	R	S
G025_C3	R	S	R	R	S	S	S	S	R	S	R	R	S	R	S	S	S
G025_B4	R	S	R	R	S	S	S	S	R	S	R	R	R	S	S	S	S
G025_B5	R	S	R	R	R	R	R	S	R	R	R	R	R	R	R	S	S
G025_B6	R	R	R	R	S	S	S	S	R	S	R	R	R	S	S	S	S
G025_B7	R	S	R	R	S	S	S	S	R	S	R	R	R	S	S	S	S
G025_B8	R	R	R	R	S	S	S	S	R	S	R	R	S	R	S	S	S
G025_B9	R	S	R	R	S	R	R	S	R	R	R	R	R	R	R	S	S
G025_B1	R	S	R	R	R	R	R	S	R	R	R	R	R	R	R	R	R
G025_B2	R	S	R	R	R	R	R	S	R	R	R	R	R	R	R	R	R
G026_S3	R	S	R	R	R	S	R	S	R	R	R	R	R	R	R	R	R
G026_S4	R	S	R	R	R	S	R	S	R	R	R	R	R	R	R	S	R
G026_S5	R	S	R	R	R	R	S	S	R	S	R	R	R	R	R	S	R
G026_S6	R	S	R	R	R	S	R	S	R	R	R	R	R	R	R	R	R
G026_S7	R	S	R	R	R	S	R	S	R	R	R	R	R	R	R	S	R
G026_S8	R	S	R	R	R	R	S	S	R	S	R	R	R	R	R	S	R

G026_S9	R	S	R	R	R	S	R	S	R	R	R	R	R	R	R	R	R
G026_S10	R	S	R	R	R	S	R	S	R	R	R	R	R	R	R	R	R
G026_C1	R	S	R	R	R	R	R	S	R	S	R	R	R	R	R	S	R
G026_C2	R	S	R	R	R	R	S	S	R	S	R	R	S	S	R	S	S
G026_C3	R	S	R	R	R	R	S	S	R	S	R	R	S	S	R	S	R
G026_C4	R	S	R	R	R	R	S	S	R	S	R	R	S	S	R	S	S
G026_C5	R	R	R	R	S	R	R	S	R	S	R	R	S	S	R	S	S
G026_C6	R	R	R	R	S	R	S	S	R	S	R	R	S	S	R	S	S
G026_C7	R	S	R	R	S	R	S	S	R	S	R	R	S	S	R	S	S
G026_C8	R	R	R	R	S	R	R	S	R	S	R	R	S	S	R	S	S
G026_B9	R	R	R	R	S	R	S	S	R	S	R	R	S	S	R	S	S
G026_B10	R	S	R	R	R	R	S	S	R	S	R	R	S	S	R	S	R
G026_B1	R	S	R	R	R	R	S	S	R	S	R	R	S	S	R	S	R
G026_B2	R	S	R	R	R	R	R	S	R	S	R	R	R	S	R	R	R
G026_B3	R	R	R	R	R	R	R	S	R	S	R	R	R	S	R	R	S
G026_B4	R	S	R	R	R	R	R	S	R	S	R	R	R	S	R	R	S
G026_B5	R	S	R	R	R	R	R	S	R	S	R	R	R	S	R	R	R
G026_B6	R	R	R	R	S	R	S	S	R	S	R	R	S	S	R	S	S
G027_S7	R	S	R	R	R	R	S	S	R	S	R	R	S	S	R	S	R
G027_S8	R	S	R	R	R	R	R	S	R	S	R	R	R	S	R	R	R
G027_S9	R	R	R	R	S	R	R	S	R	S	R	R	R	S	R	R	S
G027_S10	R	S	R	R	S	R	R	S	R	S	R	R	R	S	R	R	S
G027_S1	S	S	R	R	S	S	S	S	S	S	S	S	S	S	S	S	R
G027_S2	R	S	R	R	R	S	R	S	S	S	R	S	S	S	S	S	S
G027_S3	R	S	R	R	R	S	R	S	S	S	R	S	S	S	S	S	S
G027_S4	S	S	S	R	S	S	S	S	R	S	R	R	R	R	R	S	S
G027_C5	R	S	R	R	R	S	R	S	R	S	R	R	R	R	R	S	S
G027_C6	S	S	S	R	S	S	S	S	R	S	R	R	R	R	R	S	S
G027_C7	R	S	R	R	R	S	R	S	S	S	R	S	S	S	S	S	S
G027_C8	S	S	S	R	S	S	S	S	R	S	R	R	R	R	R	S	S
G027_C9	S	S	R	R	S	S	S	S	S	S	S	S	S	S	S	S	R
G027_C10	R	S	R	R	R	S	R	S	S	S	R	S	S	S	S	S	S
G027_C1	S	S	S	R	S	S	S	S	R	S	R	S	R	R	R	S	S
G027_C2	S	S	S	R	S	S	S	S	S	S	R	S	S	S	S	S	R
G027_B3	S	S	S	R	S	S	S	S	S	S	S	S	R	S	S	S	S
G027_B4	R	S	R	R	R	S	R	S	R	S	R	R	R	R	R	S	S
G027_B5	R	S	R	R	R	S	R	S	S	R	R	S	S	S	S	S	S
G027_B6	R	S	R	R	R	S	R	S	S	R	R	S	R	S	S	S	S
G027_B7	S	S	S	R	S	S	S	S	S	S	R	S	S	S	S	S	R
G027_B8	S	S	S	R	S	S	S	S	R	S	R	S	R	R	R	S	S
G027_B9	R	S	R	R	R	S	R	S	R	S	R	R	R	R	R	S	S
G027_B10	R	S	R	R	R	S	R	S	S	R	R	S	S	S	S	S	S
G028_S2	S	S	S	R	S	S	S	S	S	S	R	S	S	R	S	R	S
G028_S3	S	R	S	R	S	S	S	S	S	S	R	S	S	R	S	R	S
G028_S4	R	S	S	R	R	S	R	S	R	S	R	R	S	R	R	R	S
G028_S5	S	S	S	R	S	S	S	S	S	S	R	S	S	R	S	R	S
G028_S6	S	S	S	R	S	S	S	S	S	S	R	S	S	R	S	R	S

G028_S7	S	R	S	R	S	S	S	S	S	S	R	S	S	R	S	R	S
G028_S8	R	S	R	R	R	S	R	S	R	S	R	R	R	R	R	R	S
G028_S9	S	S	S	R	S	S	S	S	S	S	R	S	S	R	S	S	S
G028_C10	S	S	S	R	S	S	S	S	S	S	R	S	S	R	S	R	S
G028_C11	S	S	S	R	S	S	S	S	S	S	R	S	S	R	S	R	S
G028_C1	R	S	R	R	R	S	R	S	S	R	R	S	S	R	S	S	S
G028_C3	S	R	S	R	S	S	S	S	S	S	R	S	S	R	S	R	S
G028_C4	S	S	S	R	S	S	S	S	R	S	S	R	S	S	R	S	R
G028_C5	R	S	R	R	R	S	R	S	R	S	R	R	R	R	R	R	S
G028_C6	S	S	S	R	R	S	S	S	S	S	R	S	S	R	R	S	S
G028_C7	S	R	S	R	S	S	S	S	R	S	R	S	S	R	S	S	R
G028_B8	S	R	S	R	S	S	S	S	S	S	R	S	S	R	S	S	S
G028_B9	S	S	S	R	S	S	S	S	S	S	R	S	S	R	S	S	S
G028_B10	S	S	S	R	S	S	S	S	R	S	S	R	S	S	R	S	R
G028_B11	S	S	S	R	S	S	S	S	S	S	R	S	S	R	S	S	S
G028_B12	S	S	S	R	S	S	S	S	R	S	S	R	S	S	R	S	R
G028_B13	S	S	S	R	S	R	S	S	R	S	R	R	R	R	R	R	S
G028_B14	R	S	S	R	R	S	R	S	R	S	R	R	S	R	R	R	S
G028_B15	S	S	S	R	S	S	S	S	S	S	S	S	S	S	S	S	S
G029_S2	S	S	S	R	S	S	S	S	S	S	S	S	S	S	S	S	S
G029_S3	S	S	S	R	S	S	S	S	S	S	R	S	S	R	S	S	S
G029_S4	S	S	S	R	S	S	S	S	S	S	S	S	S	S	S	S	S
G029_S5	R	S	S	R	R	S	R	S	R	S	R	R	S	R	R	R	S
G029_S6	S	S	S	R	S	S	S	S	S	S	R	S	S	R	S	S	S
G029_S7	S	S	S	R	S	S	S	S	S	S	S	S	S	S	S	S	S
G029_S8	S	S	S	R	S	S	S	S	S	S	S	S	S	S	S	S	S
G029_S9	S	S	S	R	S	S	S	S	S	S	S	S	S	S	S	S	S
G029_S10	S	S	S	R	S	S	S	S	S	S	R	S	S	R	S	S	S
G029_S3	R	S	S	R	R	S	R	S	R	S	R	S	S	S	S	S	S
G029_C4	R	S	R	R	R	S	R	S	R	R	R	R	S	R	R	S	S
G029_C5	S	S	S	R	S	S	S	S	S	S	S	S	S	S	S	S	S
G029_C6	R	S	S	R	R	S	R	S	R	S	R	S	S	S	S	S	S
G029_C7	R	S	S	R	R	S	R	S	R	S	R	S	S	S	S	S	S
G029_C8	S	R	S	R	S	S	S	S	R	S	R	R	S	R	R	R	R
G029_C9	S	R	S	R	S	S	S	S	R	S	R	R	S	R	R	R	R
G029_C10	R	S	S	R	R	S	R	S	R	S	R	S	S	S	S	S	S
G029_C11	R	S	R	R	R	S	R	S	R	R	R	R	S	R	R	S	S
G029_C12	S	S	S	R	S	S	S	S	S	S	S	S	S	S	S	S	S
G029_C2	R	S	R	R	R	S	R	S	R	R	R	R	S	R	R	S	S
G029_C3	S	S	S	R	S	S	S	S	S	S	S	S	S	S	S	S	S
G029_B4	R	S	S	R	R	S	R	S	S	S	R	S	S	S	S	S	S
G029_B5	R	S	R	R	R	S	R	S	R	S	R	R	S	R	R	S	S
G029_B6	S	S	R	R	R	S	R	S	R	S	R	R	S	R	R	S	S
G029_B7	S	S	S	R	S	S	S	S	S	S	S	S	S	S	S	S	S
G029_B8	S	S	R	R	R	S	R	S	R	S	R	R	S	R	R	S	S
G029_B9	S	S	S	R	S	S	S	S	S	S	S	S	S	S	S	S	S
G029_B10	S	R	S	R	S	S	S	S	R	S	R	R	S	R	R	R	R

G030_S11	S	S	S	R	R	S	S	S	R	S	R	R	S	S	R	S	R
G030_S12	S	R	S	R	S	S	S	S	S	S	R	S	S	S	S	S	S
G030_S1	S	S	S	R	S	S	S	S	S	S	R	S	S	S	S	S	S
G030_S2	S	R	S	R	R	S	S	S	S	S	R	S	R	R	S	R	S
G030_S3	S	S	S	R	S	S	S	S	S	S	R	S	S	S	S	R	S
G030_S4	S	R	S	R	S	S	S	S	R	S	R	R	R	R	R	S	R
G030_S5	R	S	R	R	R	S	R	S	S	S	R	S	S	S	S	S	S
G030_S6	S	S	R	R	R	S	R	S	R	S	R	R	R	R	R	S	R
G030_C7	S	S	S	R	S	S	S	S	R	S	S	R	R	R	R	S	S
G030_C8	S	S	S	R	S	S	S	S	R	S	R	R	R	R	R	S	S
G030_C9	S	S	S	S	S	S	S	S	R	S	S	R	R	R	R	S	S
G030_C10	S	S	S	R	S	S	S	S	R	S	S	R	R	R	R	S	S
G030_C2	S	S	S	S	S	S	S	S	S	S	S	S	S	R	S	R	S
G030_C3	S	S	S	R	R	S	S	S	R	S	R	R	S	R	R	S	R
G030_C4	S	S	S	R	R	S	R	S	R	S	R	R	S	R	R	S	R
G030_C5	S	S	S	R	R	S	S	S	R	S	R	R	S	R	R	S	R
G030_B6	S	S	S	R	R	S	R	S	R	S	R	R	S	R	R	S	R
G030_B7	S	S	S	R	R	S	S	S	R	S	R	R	S	R	R	S	R
G030_B8	S	S	S	S	R	S	S	S	S	S	S	S	S	R	S	R	S
G030_B9	S	S	S	R	R	S	S	S	R	S	R	R	S	R	R	S	R
G030_B10	S	S	S	S	S	S	S	S	S	S	S	S	S	R	S	R	S
G030_B11	S	R	S	R	R	S	S	S	R	S	R	R	S	R	R	S	R
G030_ B1	R	S	R	R	R	S	S	S	S	R	R	R	R	S	R	S	S
G030_ B2	R	S	R	R	R	S	S	S	R	R	R	R	R	S	R	S	S
G030_ B3	R	S	R	R	R	R	R	S	R	R	R	R	R	S	R	R	R
G031_ S4	R	S	R	R	R	S	S	S	R	R	R	R	R	S	R	S	S
G031_ S5	R	R	R	R	R	R	S	S	R	R	R	R	R	R	R	S	S
G031_ S6	R	R	R	R	R	R	S	S	R	R	R	R	R	R	R	S	S
G031_ S7	R	S	R	R	R	R	R	S	R	R	R	R	R	S	R	R	R
G031_ S8	R	S	R	R	R	S	S	S	S	R	R	R	R	S	R	S	S
G031_ S9	R	S	R	R	R	S	S	S	R	R	R	R	R	S	R	S	S
G031_S10	R	S	R	R	R	S	S	S	R	R	R	R	R	S	R	S	S
G031_S2	R	R	R	R	R	R	S	S	R	S	R	R	R	R	R	S	R
G031_C3	R	R	R	R	R	R	S	S	R	R	R	R	R	R	R	S	S
G031_C4	R	S	R	R	R	S	R	S	R	R	R	R	R	R	R	R	S
G031_C5	R	R	R	R	R	R	S	S	R	S	R	R	S	R	R	S	R
G031_C6	R	R	R	R	R	R	S	S	R	S	R	R	R	R	R	S	R
G031_C7	R	R	R	R	R	R	S	S	R	S	R	R	R	R	R	S	R
G031_C8	R	S	R	R	R	S	R	S	R	R	R	R	R	R	R	R	S
G031_C9	R	R	R	R	R	R	S	S	R	S	R	R	S	R	R	S	R
G031_C10	R	S	R	R	R	S	R	S	R	R	R	R	R	R	R	R	S
G031_B11	R	R	R	R	R	R	S	S	R	S	R	R	R	R	R	S	R
G031_B1	R	S	R	R	R	S	R	S	R	R	R	R	S	R	R	S	R
G031_B2	R	S	R	R	R	S	R	S	R	R	R	R	S	R	R	S	R
G031_B3	R	R	R	R	R	S	S	S	R	R	R	R	R	R	R	R	R
G031_B4	R	S	R	R	R	S	S	S	R	R	R	R	R	R	R	S	R
G031_B5	R	R	R	R	R	R	R	S	R	R	R	R	R	S	R	R	R

G031_B6	R	S	R	R	R	S	R	S	R	R	R	R	S	R	R	S	R
G031_B7	R	S	R	R	R	S	R	S	R	R	R	R	S	R	R	S	R
G031_B8	R	S	R	R	R	S	R	S	R	R	R	R	S	R	R	S	R
G031_B9	R	S	R	R	R	S	S	S	R	R	R	R	R	R	R	S	R

G0x – number of the farm; _C – poultry litter; _S – cloacal swab; _B – beetles; AMC – amoxicillin + clavulanic acid; AMP – ampicillin; ATM – aztreonam; CTX – cefotaxime; CFO – cefoxitin; CFZ – cefazolin; CIP – ciprofloxacin; CLO – chloramphenicol; ENR – enrofloxacin; FEP – cefepime; FLF – florfenicol; FOS – fosfomycin; GEN – gentamicin; IPM – imipenem; NAL – nalidixic acid; SUT – sulfamethoxazole/trimethoprim; TET – tetracycline.
